# The roles, activities and impacts of middle managers who function as knowledge brokers to improve care delivery and outcomes in healthcare organizations: a critical interpretive synthesis

**DOI:** 10.1186/s12913-021-07387-z

**Published:** 2022-01-02

**Authors:** Faith Boutcher, Whitney Berta, Robin Urquhart, Anna R. Gagliardi

**Affiliations:** 1 Baycrest Health Sciences, 3560 Bathurst Street, Toronto, Ontario M6A 2E1 Canada; 2 Institute of Health Policy, Management and Evaluation, University of Toronto, Health Sciences Building Suite 425, 155 College Street, Toronto, Ontario M5T 3M6 Canada; 3 Department of Community Health and Epidemiology, Dalhousie University, Room 413, 5790 University Avenue, Halifax, Nova Scotia B3H 1V7 Canada; 4 University Health Network, 13EN-228, 200 Elizabeth Street, Toronto, Ontario M5G 2C4 Canada

**Keywords:** Middle managers, Knowledge brokers, Critical interpretive synthesis

## Abstract

**Background:**

Middle Managers (MMs) are thought to play a pivotal role as knowledge brokers (KBs) in healthcare organizations. However, the role of MMs who function as KBs (MM KBs) in health care is under-studied. Research is needed that contributes to our understanding of how MMs broker knowledge in health care and what factors influence their KB efforts.

**Methods:**

We used a critical interpretive synthesis (CIS) approach to review both qualitative and quantitative studies to develop an organizing framework of how MMs enact the KB role in health care. We used compass questions to create a search strategy and electronic searches were conducted in MEDLINE, CINAHL, Social Sciences Abstracts, ABI/INFORM, EMBASE, PubMed, PsycINFO, ERIC and the Cochrane Library. Searching, sampling, and data analysis was an iterative process, using constant comparison, to synthesize the results.

**Results:**

We included 41 articles (38 empirical studies and 3 conceptual papers) that met the eligibility criteria. No existing review was found on this topic. A synthesis of the studies revealed 12 MM KB roles and 63 associated activities beyond existing roles hypothesized by extant theory, and we elaborate on two MM KB roles: 1) convincing others of the need for, and benefit of an innovation or evidence-based practice; and 2) functioning as a strategic influencer. We identified organizational and individual factors that may influence the efforts of MM KBs in healthcare organizations. Additionally, we found that the MM KB role was associated with enhanced provider knowledge, and skills, as well as improved organizational outcomes.

**Conclusion:**

Our findings suggest that MMs do enact KB roles in healthcare settings to implement innovations and practice change. Our organizing framework offers a novel conceptualization of MM KBs that advances understanding of the emerging KB role that MMs play in healthcare organizations. In addition to roles, this study contributes to the extant literature by revealing factors that may influence the efforts and impacts of MM KBs in healthcare organizations. Future studies are required to refine and strengthen this framework.

**Trial registration:**

A protocol for this review was not registered.

**Supplementary Information:**

The online version contains supplementary material available at 10.1186/s12913-021-07387-z.

Contributions to the literature

- MMs may play an important KB role in healthcare organizations.
- Additional support for the MM KB role may help enhance quality of care in healthcare settings.
- An improved understanding of MM KBs will contribute to this nascent area of inquiry in health care.

## Background

Health systems are under increasing pressure to improve performance including productivity, quality of care, and efficiency in service delivery. To promote optimal performance, health systems hold healthcare organizations such as hospitals accountable for the quality of care they provide through accountability agreements tied to performance targets [1, 2]. Despite such incentives, healthcare organizations face considerable challenges in providing high-quality care and research continues to show that the quality of hospital-based care is less than ideal [3–5]. Some researchers contend that this is attributed, in part, to the challenges that healthcare organizations face when integrating new knowledge into practice. Some challenges include dedicating sufficient resources to adopt or implement evidence-informed innovations that enhance service delivery and optimize patient health and outcomes [6].

Healthcare organizations use knowledge translation (KT) approaches to promote the use of evidence-based practices intended to optimize quality of care. The use of knowledge brokers (KBs) is one such approach. KBs are defined as the human component of KT who work collaboratively with stakeholders to facilitate the transfer and exchange of knowledge in diverse settings, [7–9]. KBs that facilitate the use of knowledge between people or groups have been referred to as opinion leaders, facilitators, champions, linking agents and change agents whose roles can be formal or informal [10, 11]. These “influencer” roles are based on the premise that interpersonal contact improves the likelihood of behavioral change associated with use or adoption of new knowledge [12]. Research shows that KBs have had a positive effect on increasing knowledge and evidence-based practices among clinicians in hospitals, and on advocating for change on behalf of clinicians to executives [13–15]. However, greater insight is needed on how to equip and support KBs, so they effectively promote and enable clinicians to use evidence-based practices that improve quality of care [13, 16, 17].

Middle managers (MMs) play a pivotal role in facilitating high quality care and may play a brokerage role in the sharing and use of knowledge in healthcare organizations [18, 19]. MMs are managers at the mid-level of an organization supervised by senior managers, and who, in turn, supervise frontline clinicians [20]. MMs facilitate the integration of new knowledge in healthcare organizations by helping clinicians appreciate the rationale for organizational changes and translating adoption decisions into on-the-ground implementation strategies [18, 19]. Current research suggests that MMs may play an essential role as internal KBs because of their mid-level positions in healthcare organizations. Some researchers have called for a deeper understanding of the MM role in knowledge brokering, including how MMs enact internal KB roles [16–19, 21].

To this end, further research is needed on who assumes the KB role and what they do. Prior research suggests that KBs may function across five key roles: knowledge manager, linking agent, capacity builder, facilitator, and evaluator, but it is not clear whether these roles are realized in all healthcare settings [7, 21, 22]. KBs are often distinguished as external or internal to the practice community that they seek to influence, and most studies have focused on external KBs with comparatively little research focused on the role of internal KBs [7, 9, 17, 23, 24]. To address this gap, we will focus on internal KBs (MMs) who hold a pivotal position because their credibility and detailed knowledge of local context allows them to overcome the barriers common to external KBs. One such barrier is resistance to advice from external sources unfamiliar with the local context [25].

With respect to what KBs do, two studies explored KB roles and activities, and generated frameworks that describe KB functions, processes, and outcomes in health care [7, 22]. However, these frameworks are not specific to MMs and are limited in detail about KB roles and functions. This knowledge is required by healthcare organizations to develop KB capacity among MMs, who can then enhance quality of care. Therefore, the focus of this study was to synthesize published research on factors that influence the KB roles, activities, and impact of MMs in healthcare settings. In doing so, we will identify key concepts, themes, and the relationships among them to generate an organizing framework that categorizes how MMs function as KBs in health care to guide future policy, practice, and research.

## Methods

### Approach

We used a critical interpretive synthesis (CIS) to systematically review the complex body of literature on MM KBs. This included qualitative, quantitative, and theoretical papers. CIS offers an iterative, dynamic, recursive, and reflexive approach to qualitative synthesis. CIS was well-suited to review the MM KB literature than traditional systematic review methods because it integrates findings from diverse studies into a single, coherent framework based on new theoretical insights and interpretations [26, 27]. A key feature that distinguishes CIS from other approaches to interpretive synthesis is the critical nature of the analysis that questions the way studies conceptualize and construct the topic under study and uses this as the basis for developing synthesizing arguments [26]. We ensured rigor by complying with the Enhancing Transparency in Reporting the Synthesis of Qualitative Research (ENTREQ) criteria (Additional file 1) and other criteria of trustworthiness [28, 29]. We did not register a protocol for this review.

### Search

With a medical librarian, we developed a search strategy (Additional file 2) that complied with the evidence-based checklist for peer review of electronic search strategies [30]. We included Medical Subject Headings and keywords that captured the concepts of MMs (e.g., nurse administrator, manager), explicit or non-explicit KB roles (e.g., diffusion of innovation, dissemination, broker, and facilitator), evidence-based practice (e.g., knowledge, evidence) and setting (e.g., hospital, healthcare, or health care). We searched MEDLINE, CINAHL, Social Sciences Abstracts, ABI/INFORM, EMBASE, PubMed, PsycINFO, ERIC, and the Cochrane Library from January 1, 2001, to August 14, 2020. We searched from 2001 onward because the field of KT did not substantially investigate KBs until 2001 [7, 21]. We reviewed the reference lists of eligible articles for additional relevant studies not identified by searches. As is typical of CIS, this was an iterative process allowing search terms to be expanded to optimize search results [26, 31].

### Eligibility

We generated eligibility criteria based on the PICO framework (population, intervention, comparisons, and outcomes) (Additional file 3). Populations refer to MMs functioning as KBs in hospitals or other healthcare settings but did not necessarily use those labels. Because the MM literature is emergent, we included settings other than hospitals (e.g., public health department, Veteran Affairs Medical Centres). We included studies involving clinical and non-clinical administrators, managers, directors, or operational leaders if those studies met all other inclusion criteria. The intervention of interest was how MM KBs operated in practice for the creation, use and sharing of knowledge, implementation of evidence-based practice(s), or innovation implementation. Study comparisons may have evaluated one or more MM KB roles, approaches and associated barriers, enablers and impacts alone or in comparison with other types of approaches for the sharing or implementation of knowledge, evidence, evidence-based practices, or innovations. Outcomes included but were not limited to MM KB effectiveness (change in knowledge, skills, policies and/or practices, care delivery, satisfaction in role), behaviors, and outcomes. Searches were limited to English language quantitative, randomized, or pragmatic controlled trials, case studies, surveys, quasi-experimental, qualitative, or mixed methods studies and conceptual papers. Systematic reviews were not eligible, but we screened references for additional eligible primary studies. Publications in the form of editorials, abstracts, protocols, unpublished theses, conference proceedings were not eligible.

### Screening

FB and ARG independently screened 50 titles and abstracts according to the eligibility criteria and compared and discussed results. Based on discrepancies, they modified the eligibility criteria and discussed how to apply them. Thereafter, FB screened all remaining titles, and discussed all uncertainties with ARG and the research team. FB retrieved all potentially eligible articles. FB and ARG independently screened a sample of 25 full-text articles, and again discussed selection discrepancies to further standardize how eligibility criteria were applied. Thereafter, FB screened all remaining full-text items.

### Quality appraisal

We employed quality appraisal tools relevant to different research designs: Standards for Reporting Qualitative Research (SRQR) [32], the Good Reporting of a Mixed Methods Study (GRAMMS) tool [33], Critical Appraisal of a Questionnaire Study [34], Revised Standards for Quality Improvement Reporting Excellence (SQUIRE 2.0) tool [35], and the Critical Appraisal Checklist for Quasi-Experimental Studies [36]. FB and ARG independently assessed and compared the quality of a sample of seven studies each. Thereafter, FB assessed the quality of the remaining 24 studies.

### Data extraction

We developed a data extraction form to extract information on study characteristics (date of publication, country, purpose, research design) and MM KB characteristics, roles, activities, enablers, barriers, and impacts. To pilot test data extraction, FB and ARG independently extracted data from the same 25 articles, then compared results and discussed how to refine data extraction. Thereafter, FB extracted data from remaining articles, which was independently checked by ARG, and then reviewed by the research team.

### Data analysis

FB and ARG conducted an initial reading and coding of a sample of articles independently. Codes were assigned to significant elements of data within the results and conclusions sections of the eligible articles and grouped into relevant categories with shared characteristics and organized into preliminary themes. This was an iterative process that involved ongoing consultation with the research team, who provided feedback on the codes and themes.

We created a matrix of MM KB roles and activities from extant MM and KB theory [7, 18, 22, 37] and deductively mapped themes from included studies with the matrix to help inform the analysis and interpretation of our findings. As per CIS methodology, we developed an integrative grid (matrix table) where themes pertaining to MM KB roles and activities formed columns, and themes mapped to those roles/activities from individual studies formed rows [31]. The grid helped us integrate the evidence across studies and explore relationships between concepts and themes to inductively develop synthetic constructs [31, 38]. Using a constant comparative approach, we critiqued the synthetic constructs with the full sample of papers to identify conceptual gaps in the available evidence in relation to our aims, and to ensure that the constructs were grounded in the data [31, 38]. Our interpretive reflections on MM KB roles, activities, factors, and impacts led us to develop “synthetic arguments” and we used the arguments to structure our findings (attributes, roles, activities, impacts, enablers, barriers) in an organizing framework to capture our interpretation of how MMs function as KBs in healthcare organizations. We used NVivo 12 software to assist with data analysis.

## Results

### Search results

**Fig. 1:**
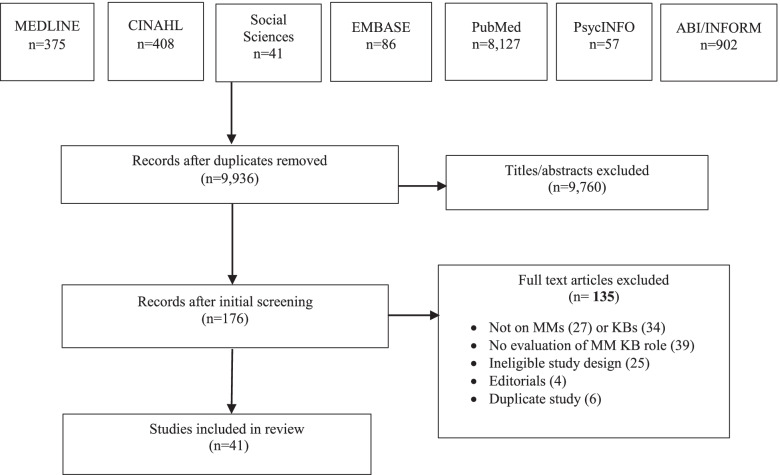
PRISMA flow diagram

The initial search yielded 9936 articles. Following removal of duplicates, 9760 titles were not eligible, and 176 items were retrieved as potentially relevant. Of those, 135 were excluded because the study design was ineligible (25), they did not examine MMs (27) or MM KBs (34), were not focused on the evaluation of an MM KB role (39), were editorials (4), or the publication was a duplicate (6). We included 41 articles for review (Fig. 1 PRISMA flow diagram). Additional file 4 includes all data extracted from included studies.

### Study characteristics

Eligible articles were published between 2003 and 2019. Three (7.3%) were conceptual and 38 (92.7%) were empirical studies. Conceptual articles discussed MM and KB theoretical constructs. Table 1 summarizes study characteristics. Studies examined the impacts of change efforts (47.3%), barriers to practice change (34.2%), and evaluation of KB interventions (18.4%). Most were qualitative (52.6%) and conducted in the United States (36.8%). Of study participants (34.2%) were MMs. In most studies, participants were nurses (63.1%) or allied health (13.2%) and based in hospitals (68.4%). Otherwise, (31.6%) were based in public health or occupational health departments, primary health care centers, Veterans Affairs Medical Centres, community care, and a senior’s care facility.

**Table 1:** Study Characteristics

Participants	n (%)
MMs	13 (34.2)
MMs & hospital staff or senior leaders	25 (65.8)
**Clinical Background**	
Nurses	24 (63.1)
Allied Health	5 (13.2)
Not specified	9 (23.6)
**Study Design**	
Qualitative	20 (52.6)
Mixed Methods	8 (21.1)
Quasi-experimental	1 (2.6)
Survey	6 (15.7)
Program Evaluation	3 (7.9)
**Country**	
Australia	4 (10.5%)
Canada	12 (31.5%)
UK	5 (13.2%)
USA	14 (36.8%)
Sweden	2 (5.2%)
Taiwan	1 (2.6%)

### Quality assessment findings

A critical analysis of the included studies revealed issues related to research design, varying from data collected from heterogeneous healthcare settings and diverse types of MMs to the type of analyses completed (e.g., qualitative, mixed methods), to the strength of conclusions drawn from a few studies’ results (e.g., correlational, or causal). Fifteen (39.5%) studies met the criteria for quality. Twenty-three (60.5%) studies had minor methodological limitations (e.g., no research paradigm identified in qualitative studies, and mixed methods studies did not describe the integration of the two methods) (Additional file 5). These methodological flaws did not warrant exclusion of any studies as they provided relevant insights regarding the emerging framework.

### MM KB attributes

Seven (18.4%) studies described MM KB attributes (Table 2). Of those, 4 (10.5%) identified MM attributes, 2 (5.2%) identified KB attributes, and 1 (2.6%) identified nurse knowledge broker attributes. MM KBs were described as confident, enthusiastic, and experienced with strong research skills [41, 45]. They were also responsive and approachable, with an understanding of the complexity of an innovation and the organizational context [42–44].

**Table 2:** MM KB Attributes

Study	Role	MM KB Attributes
Bullock 2012 [39]	MM Fellow	• Willing to learn and contribute to research • Engaging • Proactive • Ongoing connection with workplace and professional colleagues to exchange knowledge and insights
Donahue 2013 [40]	MM	• Visionary
Kakyo 2017 [41]	MM	• Professional • Enthusiastic • Expert skills in managing resources
Kitson 2011 [42]	MM	• Confident • Knowledgeable • “Can do” attitude • Able to work effectively with teams • Understands the complexity of the innovation task
Schreiber 2015 [43]	KB	• Strong understanding of clinical/organizational contexts • Strong research skills • Enthusiastic • Accessible
Traynor 2014 [44]	KB	• Expert in research methodology • Approachable and patient • Comfortable dealing with people at multiple levels • Trustworthy • Flexible • Strong communication skills • Knowledgeable about evidence-informed decision making and information management • Able to pick up new knowledge quickly
Catallo 2015 [45]	Nurse KB	• Experienced in research methods • Credible clinical expert • Accountable and trustworthy • Culturally compatible

### MM KB roles and activities

Table 3 summarizes themes pertaining to roles and activities. A total of 63 activities were grouped in the following 12 MM KB roles: (1) gather data, (2) coordinate projects, (3) monitor and evaluate the progress of a project, (4) adjust implementation to organizational context, (5) disseminate information, (6) facilitate networks, (7) bridge the evidence-to-practice gap, (8) engage stakeholders, (9) convince others of the need for, and benefit of a project, (10) coach staff, (11) provide tools and resources and (12) function as a strategic influencer. Roles did not differ among MM KBs in hospital and non-hospital settings.

**Table 3:** Themes representing MM KB roles and activities

MM KB Roles	Corresponding MM KB activities	Illustrative Quote
Gather data	• Conduct needs assessments, literature searches • Obtain and diffuse information	“MMs use innovative electronic tools and visual data in order to convey real-time information to their staff” [46].
Coordinate projects	• Coordinate research activities • Apply for grants and other funding • Prepare documents, checklists • Develop tools and algorithms • Primary and co-investigators in research projects	“They were responsible for associated administrative tasks such as developing recruitment processes and interview schedules” [47].
Monitor and evaluate progress of project	• Hold meetings with teams to identify what was working and not • Monitor unit performance • Use their clinical expertise and contextual understanding to adapt materials, prioritize QI activities • Evaluate performance • Provide thoughtful feedback on performance • Initiate improvements	“MMs reported back to senior management on implementation progress and issues” [48].
Adjust implementation of project to organizational context	• Align with organization strategy, mission, vision, values • Adapt information to local context • Use daily conversations to translate the strategic plan • Integrate changes into other initiatives	“The two strategies used most commonly to adapt change to the organizational context were exploring, auditing, and monitoring best practices, and policy and documentation changes to incorporate guideline recommendations” [49]
Disseminate Information	• Present data • Attend shift report • Initiate meetings • Communicate professional updates • Use visual/verbal reminders • Facilitate staff access to evidence	“MMs frequent updates and guidance through emails, weekly briefings and attendance at shift handover was crucial” [50].
Facilitate networks	• Open doors because of networks • Engage key informants and knew gatekeepers • Network • Make influential people aware of KT issues • Use social capital to develop relationships • Locate influential partners	“Manager fellows helped by being on the inside and knowing gatekeepers and key informants and being able to engage them” [39]
Bridge the evidence-to-practice gap	• Boundary spanner • Bridge management’s requirements and patients’ needs • Link Quality Improvement teams with Senior Management • Lateral integrator • Link between researchers and staff • Link individuals and groups	“Some manager fellows were particularly valued by the research team because they also had specific knowledge or access to professional networks” [39].
Engage Stakeholders	• Use strategies to engage staff • Engage staff in developing and meeting targets • Facilitate conversations and communication • Mediate between strategy and day to day activities • Measure performance and engage in frontline activities	“MMs influence facilitating organizational change may stem from overseeing team activities, mediating between organizational strategy and day-to day activities of staff and helping interpret information in a way that is relevant to each member of the team” [40].
Convince others of the need for, and benefit of a project	• Promote and advocate the program • Be persuasive at all committee levels • Justify the innovation • Encourage staff to use those innovations consistently and effectively • Give rationale for change • Establish trust and rapport • Reinforce initiative/expectations • Hold ongoing conversations team to ensure “everyone was on board”	“The ongoing conversations the unit managers had with the project leads, clinicians, and staff ensured that “everyone was on board” for the local QI efforts. These informal conversations are important as they help clinicians perceive benefits of the improvement, promote collaboration, and emphasize accountability to ensure the successful implementation of local QI efforts” [51].
Coach staff	• Coach and mentor individual staff • Coach and mentor teams • Develop and educate staff • Act as a resource to help staff • Facilitate individual capacity development in evidence-informed decision making	“I love when I am able to interact and mentor my staff, resulting in positive outcomes for the unit. That is when I am more energized and find the most job satisfaction” (Manager Participant) [52].
Provide tools and resources	• Provide staff with the tools to implement the innovation • Provide operational support – resources, tools, training of staff • Procure organizational resources • Provide funds for training and supplies • Find creative ways to overcome barriers	“Managers tried to provide flexibility in schedules to ensure that clinicians, predominately nurses, had sufficient time and space to manage clinical and QI work” [51]
Function as strategic influencer	• Accountable 24/7 for their care areas and patient safety • Responsible for large number of employees and regulatory compliance • Lead teams • Members of decision-making groups for strategic planning • Authoritative contact for initiatives	“MMs can capitalize on their unique position between upper and lower levels in the organization and engage in ambidextrous learning that is critical to implementing and sustaining radical change” [53].

Table 4 summarizes the frequency of each of the 12 MM KB roles across included studies. The two most common MM KB roles were *to monitor and evaluate the progress of a project* (14, 36.8%) [40, 41, 47–51, 54, 57, 60, 63–66] and *to convince others of the need for, and benefit of a project* (12, 31.6%) [46–48, 50, 51, 55, 58, 61, 64–67]. For example, MM KBs played an important role in monitoring the progress of projects to evaluate and reinforce practice change [41, 50]. To convince others of the need for, and benefit of a project and to promote staff buy-in, they held ongoing conversations with staff to help them understand the rationale for change, reinforce the message, and encourage staff to consistently maintain the innovations on their units [46, 48, 66]. The least common MM KB role was project coordination (4, 10.5%) [39, 47, 48, 56].

**Table 4:** Frequency of 12 MM KB roles in included studies

MM KB Roles	Study citations	n (%)
Gather data	[39, 44, 47, 48, 54–56]	7 (18.4)
Coordinate projects	[39, 47, 48, 56]	4 (10.5)
Disseminate Information	[37, 43, 47, 50, 51, 54, 56–59]	10 (26.3)
Adjust information to organizational context	[37, 39, 41, 46, 48–50, 52, 58, 60, 61]	11 (28.9)
Bridge the evidence-to-practice gap	[39, 43, 47, 54, 58, 62]	6 (15.8)
Facilitate networks	[39, 45, 49, 56, 57, 59–63]	10 (26.3)
Engage stakeholders	[37, 40, 41, 50, 57, 63, 64]	7 (18.4)
Provide tools and resources	[46, 48, 51, 59, 61, 63]	6 (13.2)
Coach staff	[44, 45, 49–52, 57, 59, 62, 63, 65]	11 (28.9)
Monitor and evaluate	[40, 41, 47–51, 54, 57, 60, 63–66]	14 (36.8)
Convince others of the need for/benefit of a project	[46–48, 50, 51, 55, 58, 61, 64–67]	12 (31.6)
Function as strategic influencer	[39, 48, 52, 56, 62, 65, 68]	7 (18.4)

NOTE: Roles were counted once per study; some studies reported multiple roles

Several of the identified MM KB roles aligned with five KB roles in prior published frameworks [7, 22] and MM role theory [18, 37] (Table 5). For example, 31 (81.6%) studies described MM KB roles of *gather data, project coordination, disseminate information*, and *adjust implementation to organizational context*, which aligned with the roles and activities of a KB knowledge manager. Twenty-nine (76.3%) studies described the MM KB roles of *provide tools and resources, convince others of the need for and benefit of a project,* and *coach staff*, which aligned with the roles and activities of a KB capacity builder. We found overlap between the MM KB roles and the four hypothesized roles in MM role theory: (1) disseminate and obtain information, (2) adapt information and the innovations, (3) mediate between strategy and day to day activities, and (4) selling innovation implementation) [18, 37]. For example, we found that as capacity builders, MM KBs also mediated between strategy and day-to-day activities such as coaching staff and providing resources, and in the role of knowledge manager, MM KBs obtained, diffused, and synthesized information [18, 37].

**Table 5:** MM KB roles in included studies mapped to roles in published KB frameworks

MM KB roles in included studies	Published MM or KB roles				
	Knowledge manager	Linking agent	Capacity builder	Evaluator	Facilitator
Gather data	X				
Coordinate projects	X				
Disseminate information	X				
Adjust information to context	X				
Bridge evidence-to-practice gap		X			
Facilitate networks		X			X
Engage stakeholders		X	X		X
Provide resources and tools			X		
Coach staff			X		
Monitor and evaluate	X			X	
Convince others of need/benefit			X		X
Function as strategic influencer	X	X			X

While MM KB roles identified in included studies aligned with the five previously identified KB roles, the CIS approach we employed identified 12 distinct roles that were further characterized based on corresponding activities associated with each of the 12 roles. Therefore, while this research agrees with prior work on MM KB roles, it represents a robust framework of MM KB roles and activities by elaborating the complexity of MM KB roles and activities.

We fully described two roles compared with prior frameworks: *to convince others of the need for and benefit of a project,* and *function as a strategic influencer.* To *convince others of the need for and benefit of a project* (e.g., a quality improvement, best practice guideline implementation, or innovation), MM KBs used tactics such as role modelling their commitment, providing the rationale for the change, being enthusiastic about its adoption, offering positive reinforcement, and providing emotional support [47, 50, 58]. The role of *strategic influencer* featured in 7 (18.4%) studies [39, 48, 52, 56, 62, 65, 68]. For example, MM KBs were influential at the executive level of the hospital, advocating for innovations among less involved team members and administrators, including the hospital board, were members of organizational decision-making groups for strategic planning, and served as an authoritative contact for initiatives.

### Factors that influence MMs knowledge brokering

Table 6 summarizes the enablers and barriers of MM KB roles and activities, organized as individual or organizational factors. We identified four enablers at the organizational level: senior management support, availability of resources, engaged staff, and alignment to strategy. The most common was senior management support, featured in 12 (32.0%) studies. We found that senior management support enhanced the commitment of MM KBs to innovation implementation [16, 17, 19, 44, 45, 52, 61, 63, 66–70]. For example, senior managers empowered and supported MM KBs to make decisions by ensuring that the necessary structures and resources were in place, and by conveying that the implementation was an organizational priority [66, 68]. We identified three individual-level facilitators: training and mentorship, personal attributes, and experience in the MM role. The most common facilitator was training and mentorship, featured in 8 (21.1%) studies. We found that training and mentorship with more experienced managers was important to the success of MM KBs and their projects, especially if they were new to their role [16, 17, 19, 41, 42, 48, 54, 68].

**Table 6:** Factors that influenced MM KB roles, activities, and impacts

Level	Factors	Studies	n (%)
Enablers, organizational	Senior management support	[16, 17, 19, 44, 45, 52, 63, 66–70]	12 (32.0)
	Availability of resources (budget, staff)	[16, 17, 19, 39, 51, 60, 67, 68]	8 (21.1)
	Engaged team	[17, 19, 40, 67, 70]	5 (13.2)
	Alignment with strategy	[57, 60, 66, 67]	4 (10.5)
Enablers, individual	Training and mentorship	[16, 17, 19, 41, 42, 48, 54, 68]	8 (21.1)
	Personal attributes	[39–45]	7 (18.4)
	Experience in the MM role	[39, 42, 44, 47, 52, 57, 68]	7 (18.4)
Barriers, organizational	Lack of senior management support	[17, 19, 52, 63, 67, 70, 71]	7 (18.4)
	Lack of resources (budget, staff)	[39, 42, 44, 47, 52, 55, 57, 64, 68–71]	12 (32.0)
	Staff resistance	[39, 42, 52, 54, 57, 63, 64, 67]	8 (21.1)
	Lack of time	[17, 19, 39, 42, 44, 47, 52, 55, 57, 64, 68, 71]	12 (32.0)
Barriers, individual	Lack of training	[17, 39, 41, 42, 55, 57, 69, 71]	8 (21.1)
	Dissatisfaction with work-life balance	[42, 44, 51, 52, 57, 61, 64, 71]	8 (21.1)
	Caught in the middle	[39, 52, 55, 56, 63, 65]	6 (15.8)
	Professional boundaries	[42, 55, 57]	3 (7.9)

Studies reported more barriers (*n* = 8) than enablers (*n* = 7). We found four organizational barriers: a lack of resources, lack of senior management support, staff resistance, and a lack of time. The most common barriers were lack of resources in 12 (32.0%) studies and lack of time in 12 (32.0%) studies. A lack of resources (budget constraints, limited staff) made it challenging for MM KBs to move their projects forward [39, 42, 44, 47, 52, 55, 57, 64, 68–71]. For example, inadequate funds interfered with obtaining appropriate resources and undermined the feasibility of implementing projects [47, 55]. In addition, staffing issues created difficulty in engaging staff in project work and low staffing levels limited capacity to provide desired standards of care [42, 64]. Additionally, a lack of protected time for data collection or other project work was identified as a significant barrier to implementing projects [17, 19, 39, 42, 44, 47, 52, 55, 57, 64, 68, 71]. MM KBs also lacked the time to nurture, support and adequately coach staff [39, 55].

We identified four individual-level barriers: lack of formal training, dissatisfaction with work life balance, being caught in the middle, and professional boundaries. The most common barriers were lack of formal training (8, 21.1%) and dissatisfaction with work life balance (8, 21.1%). For example, a lack of formal training resulted in MM KBs being unprepared for managerial roles and without the knowledge and skills to promote effective knowledge brokering and knowledge transfer with end users [17, 39, 41, 42, 55, 57, 69, 71]. We also found that heavy workloads and conflicting priorities left MM KBs often dissatisfied with their work life balance and hindered their ability to successfully complete projects [42, 44, 51, 52, 57, 61, 64, 71]. For example, because of multiple responsibilities and conflicting priorities, MM KBs were often pulled away to address problems or were so absorbed by administrative tasks that they had no time to complete project responsibilities [44, 64].

### Impact on service delivery and outcomes

Eight (21.1%) studies showed that MM KBs had some impact on organizational and provider outcomes [16, 40, 43, 44, 47, 56, 62, 67]. One (2.6%) study reported that practice changes were greater when associated with higher MM leadership scores (OR 1.92 to 6.78) and when MMs worked to help create and sustain practice changes [40]. One (2.6%) study reported the impact of senior managers’ implementation of an evidence-based Hospital Elder Life Program on administrative outcomes (e.g., reduced length of stay and cost per patient), clinical outcomes (e.g., decreased episodes of delirium and reduced falls), and provider outcomes (e.g., increased knowledge and satisfaction) [67].

Two (5.3%) studies reported the impact of a Clinical Nurse Leader role on care processes at the service level in American hospitals. Benefits were evident in administrative outcomes such as RN hours per patient day (increased from 3.76 to 4.07) and in reduced surgical cancellation rates from 30 to 14%. There were also significantly improved patient outcomes in dementia care, pressure ulcer prevention, as well as ventilator-assisted pneumonia [56, 62]. One (2.6%) study reported financial savings [56].

Four (10.5%) studies reported the effect of a KB strategy on health professionals’ knowledge, skills, and practices [16, 43, 44, 47]. For example, Traynor et al. [44] found that participants who worked closely with a KB showed a statistically significant increase in knowledge and skill (average increase of 2.8 points out of a possible 36 (95% CI 2.0 to 3.6, *p* < 0.001) from baseline.

### Organizing framework of MM KBs in healthcare organizations

**Fig. 2:**
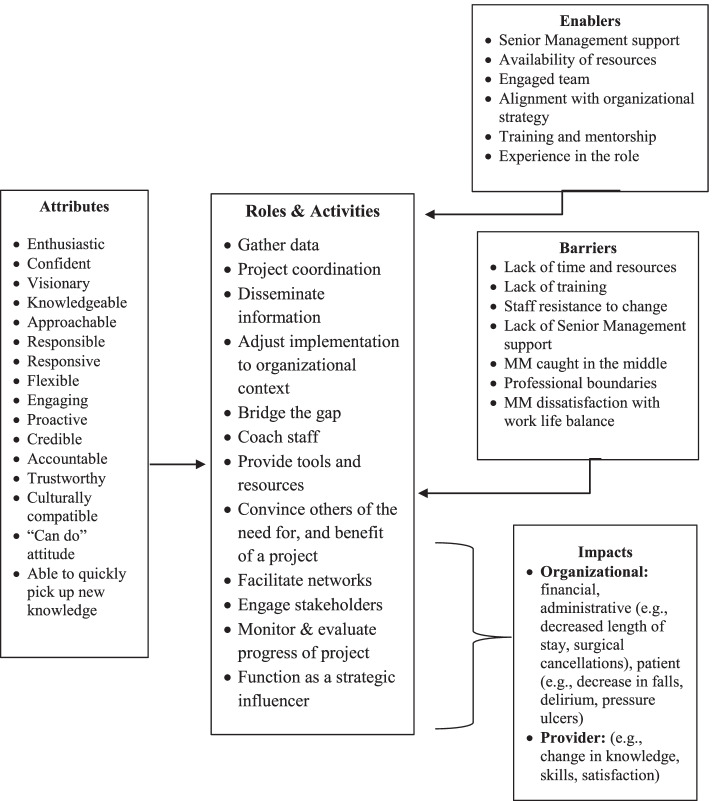
Organizing framework of MMs who function as knowledge brokers in healthcare organizations

We sought to capture the roles, activities, enablers, barriers and impacts of MM KBs across diverse healthcare settings in an organizing framework (Fig. 2 Organizing framework of MMs who function as knowledge brokers in healthcare organizations). From our interpretation of the published evidence, the findings across studies were categorized into 12 roles and 63 associated activities to represent specific ways in which MM KBs described their roles and activities during project implementation. Influencing factors were categorized into individual and organizational enablers and barriers that influence the efforts of MM KBs in healthcare organizations. While attributes were categorized as enablers, their level of importance as enablers emerged from our synthesis in how they operated in practice. The types of outcomes that we examined also varied between changes in care practice, processes, and competencies which we constructed into provider and organizational outcomes. Our emergent insights were used to construct four synthesizing arguments from the available literature: (1) *MM KBs have attributes that equip and motivate them to implement practice change and innovations in healthcare organizations,* (2) *MMs enact KB roles and activities in healthcare organizations,* (3) *enablers and barriers influence the knowledge brokering efforts of MMs in healthcare settings;* and (4) *MM KB efforts impact healthcare service delivery.* These synthesizing arguments were used to structure the organizing framework presented in Fig. 2, which depicts how MM function as KBs in healthcare organizations and their impact on service delivery.

## Discussion

We conducted a CIS to synthesize published research on factors that influence the roles, activities, and impacts of MM KBs in healthcare organizations. As per CIS, our output was an organizing framework (Fig. 2) that promotes expansive thinking about and extends knowledge of MM KBs in healthcare settings. We identified 63 activities organized within 12 distinct MM KB roles, which is far more comprehensive than any other study [7, 22]. We build on prior frameworks and characterize further the roles of strategic influencer and convincing others of the need for, and benefit of an innovation or evidence-based practice. We identified organizational and individual enablers and barriers that may influence the efforts and impact of MM KBs in health care. Of note, a key enabler was senior leadership support while a key barrier for MM KBs was a lack of formal training in project implementation. Such factors should be closely considered when looking at how to strengthen the MM KB role in practice. Furthermore, we found that the MM KB role was associated with enhanced provider knowledge and skills, as well as improved clinical and organizational outcomes.

We offer a novel conceptualization of MM KBs in healthcare organizations that has, thus far, not been considered in the literature. Our theoretical insights (summarized in Fig. 2) are an important first step in understanding how individual and organizational factors may influence how MMs enact KB roles, and the impact they have on service delivery and associated outcomes. We found that the many MM KB roles and activities corresponded to the characterization of KB roles in the literature and substantiated MM role theory. Our findings corroborate previous studies and systematic reviews by confirming that MMs function as KBs and build on the MM and KB theoretical constructs previously identified in the literature [7, 18, 21, 22, 37, 46, 48]. Building on Birken and colleagues’ theory [37], we found significant overlap between MM and KB roles and activities. Figure 2 helps to define and analyze the intersection of these roles while distinguishing MM KB roles and activities more clearly from other administrative roles.

We contend that Fig. 2 has applicability across a range of healthcare settings and may be used by hospital administrators, policymakers, service providers, and researchers to plan projects and programs. It may be used as a resource in strategic planning, to re-structure clinical programs, build staff capacity, and optimize HR practices. For example, Fig. 2 could be used as a foundation to establish goals, objectives, or key performance indicators for a new or existing clinical program; refine job postings for MM roles to encompass optimal characteristics of candidates to enable KB activities; or identify new evaluation criteria for staff performance and training gaps in existing HR practices. It could also help decision makers take on pilot projects to formalize the KB role in healthcare.

Figure 2 is intended to foster further discussion of the role that MMs play in brokering knowledge in healthcare settings. It can be modified for specific applications, although we encourage retaining the basic structure (reflecting the synthesizing arguments). For example, the factors may change depending on specific localized healthcare contexts (i.e., acute care versus long-term care, or rehabilitation). Although the use of our framework in practice has yet to be evaluated, it may be strengthened with the results of additional mixed methods studies examining MM KBs as well as quasi-experimental studies applying adapted HR practices based upon our framework. As more studies are reported in the literature, the roles, activities, factors, and outcomes can be further refined, organized, and contextualized. Figure 2 can also be used as a guide for future studies examining how MMs enact the KB role across healthcare settings and systems, disciplines, and geographic locations.

Our synthesis provides new insights into the roles of MM KBs in healthcare settings. For example, we further elucidate two MM KB roles: 1) functioning as a strategic influencer; and 2) convincing others of the need for, and benefit of an innovation or evidence-based practice. These are important roles that MM KBs enact when preparing staff for implementation and corroborate Birken et al.’s hypothesized MM role of selling innovation implementation [18, 37]. Our findings validate the organizational change literature that emphasizes the important information broker role MMs play in communicating with senior management and helping frontline staff achieve desired changes by bridging information gaps that might otherwise impede innovation implementation [37]. Our new conceptualization of how MM KBs navigate and enact their roles, and the impact they may have on service delivery and associated outcomes extends the findings of recent studies. These studies found that the role of MMs in organizational change is evolving and elements such as characteristics and context may influence their ability to facilitate organizational adaptation and lead the translation of new ideas [53, 72, 73]. However, further research is required to test and further explicate these relationships in the broader context of practice change.

Our synthesis both confirms and extends previous research by revealing organizational and individual factors that both enabled and hindered MM KBs efforts in healthcare organizations. An important organizational factor in our study was having senior management support. We found that MM KBs who had healthy supportive working relationships with their senior leaders led to project success. This support was critical because without it they experienced significant stress at being “caught in the middle” trying to address the needs of staff while also meeting the demands of senior management. Recent studies confirm our finding that senior management engagement is essential to MM KBs’ ability to implement innovations and underscores the need for senior leaders to be aware of, and acknowledge, the impact that excessive workload, competing demands, and role stress can play in their effectiveness [19, 74].

The personal attributes of MM KBs as well as their level of experience were both important factors in how they operated in practice. We identified that key attributes of MM KBs contributed to their ability to drive implementation of initiatives and enhanced staff acceptance and motivation to implement practice change [75, 76]. Our findings corroborate recent studies that highlight how the key attributes of effective champions (those that are intrinsic and cannot be taught) [77–79] may contribute to their ability to lead teams to successful implementation outcomes in healthcare organizations [80–82]. We also found that experienced MM KBs were well trained, knowledgeable, and better prepared to understand the practice context than novice MM KBs, but a lack of formal training in project implementation was an impediment for both. This emphasizes the importance of providing opportunities for professional development and training to prepare both novice and experienced MM KBs to successfully implement practice change. Our findings contribute to the growing knowledge base regarding what makes an effective MM KB. However, future research should focus on generating evidence, not only on the attributes of MM KBs, but also on how those attributes contribute to their organizational KB roles as well as the relationships among specific “attributes” and specific KB roles. More research is also needed to better understand how and what skills can be taught to boost the professional growth of MM KBs in health care.

Organizational theory and research may provide further insight into our findings and guidance for future research on the role of MM KBs in healthcare organizations. For example, the literature suggests that by increasing MMs’ appreciation of evidence-based practice, context, and implementation strategies may enhance their role in implementing evidence-based practices in healthcare organizations [18, 83, 84]. We found that MM KBs’ commitment to the implementation of an evidence-based project was influenced by the availability of resources, alignment with organizational priorities, a supportive staff and senior leadership. Extending from organizational theory and research, further investigation is needed to explore the nature of the relationship between these factors and the commitment of MM KBs to evidence-based practice implementation and subsequent outcomes.

When assessing the impact of MM KBs in hospitals, we found some evidence of changes in organizational and provider outcomes, suggesting MM KB impact on service delivery. Given that the available outcome data were limited, associational in nature, or poorly evaluated, it was challenging to identify strong thematic areas. Like our study, several systematic reviews also reported the lack of available outcome data [7, 18, 21]. This highlights an important area for research. Future research must include evaluation of the effectiveness of MM KBs and establish rigorous evidence of their impact on service delivery.

Our findings have important implications for policy and practice. MMs are an untapped KB resource who understand the challenges of implementing evidence-based practices in healthcare organizations. Both policy makers and administrators need to consider the preparation and training of MM KBs. As with other studies, our study found that providing MM KBs with opportunities for training and development may yield a substantial return on investment in terms of narrowing evidence-to-practice gaps in health care [48]. Thus, an argument can be made for recruiting and training MM KBs in health care. However, the lack of guidance on how to identify, determine and develop a curriculum to prepare MM KBs requires more research.

Our synthesis revealed numerous activities associated with 12 MM KB roles providing further insight into the MM role in healthcare settings. Our list of 63 activities (Table 2) has implications for practice. We found that MMs enact numerous KB roles and activities, in addition to their day-to day operational responsibilities, highlighting the complexity of the MM KB role. Senior leaders and administrators must acknowledge this complexity. A greater understanding of these KB roles and activities may lead to MM implementation effectiveness, to sustainable MM staffing models, and to organizational structures to support the KB efforts that many MMs are already doing informally. For example, senior leaders and administrators need to take the MM KB role seriously and explicitly include KB activities as a core function of existing MM job descriptions. To date, the KB role and associated activities are not typically or explicitly written into the formal job descriptions for MMs in healthcare settings, as their focus is primarily on operational responsibilities. A formal job description for MM KBs would improve the KB capacity of MMs by giving them the permission and recognition to implement KB-related functions. Our findings inform future research by more clearly articulating the MM KB roles and activities that may be essential to the implementation of evidence-based practice and highlights a much-needed area for future work.

Our study features both strengths and weaknesses. One strength in using CIS methodology was the ability to cast a wide net representing a range of research designs of included studies. This included studies in which MMs were required to be KBs by senior leaders or functioned explicitly as KBs. This enabled us to identify and include diverse studies that made valuable theoretical contributions to the development of an emerging framework, which goes beyond the extant theories summarized in the literature to date [18]. In contrast to prior systematic reviews of MM roles in implementing innovations [18], the CIS approach is both systematic and iterative with an interpretive approach to analysis and synthesis that allowed us to capture and critically analyze an in-depth depiction of how MMs may enact the KB role in healthcare organizations. Our synthesis also revealed numerous activities associated with the 12 identified MM KB roles. The resulting theoretical insights were merged into a new organizing framework (Fig. 2). These insights are an important first step in understanding how individual and organizational factors may influence how MMs enact KB roles, and the impact they have on service delivery.

Although CIS is an innovative method of synthesizing the literature and continues to evolve, it does have limitations. CIS has yet to be rigorously evaluated [85, 86]. While there is some precedent guiding the steps to conduct a CIS, one weakness is that CIS is difficult to operationalize. Another weakness is that the steps to conduct CIS reviews are still being refined and can lack transparency. Therefore, we used standardized, evidence-based checklists and reporting tools to assess transparency and methodological quality, and an established methodology for coding and synthesis. We provided an audit trail of the interpretive process in line with the ENTREQ guidance. Still, there was a risk of methodological bias [28, 85, 86]. Another weakness of qualitative synthesis is its inability to access first order constructs that is the full set of participants’ accounts in each study. As reviewers, we can only work with the data provided in the papers and, therefore, the findings of any review cannot assess primary datasets [31]. Study retrieval was limited to journals that are indexed in the databases that were searched. We did not search the grey literature, assuming that most empirical research on MM KBs would be found in the indexed databases. Finally, we may have synthesized too small a sample of papers to draw definitive conclusions regarding different aspects of MMs as KBs.

## Conclusion

Our study is a first step in advancing the theoretical and conceptual conversation regarding MM KBs by articulating the attributes, roles, activities, and factors influencing their efforts and impact. Through the generation of a novel organizing framework, we identify a potential combination of roles for those in MM positions who may also function as KBs in healthcare organizations. Our study is a timely contribution to the literature and offers an initial understanding of extant evidence of the KB role MMs play in health care. Our framework has utility for policymakers, administrators, and researchers to strengthen the MM role and, ultimately, improve quality of care.

## Supplementary Information

**Additional file 1.** ENTREQ checklist

**Additional file 2.** Search strategy

**Additional file 3.** Eligibility criteria

**Additional file 4.** Data extraction form for eligible studies

**Additional file 5.** Quality appraisal tools and findings

## Data Availability

All data generated or analyzed during this study are included in this published article and its supplementary information files.

## Abbreviations

MM: Middle Manager
KB: Knowledge Broker
MM KBs: Middle managers who function as Knowledge brokers
KT: Knowledge Translation
CIS: Critical Interpretive Synthesis
QI: Quality Improvement
ENTREQ: Enhancing Transparency in Reporting the Synthesis of Qualitative Research

## References

[1] Berenson RA, Rice Y (2015). Beyond measurement and reward: methods of motivating quality improvement and accountability. Health Serv Res.

[2] Nunes R, Rego G, Brandao C (2009). Healthcare regulation as a tool for public accountability. Medical Healthcare & Philosphy.

[3] Grimshaw JM, Eccles M, Lavis JN, Hill SJ, Squires JE (2012). Knowledge translation of research findings. Implement Sci.

[4] McGlynn EA, Asch SM, Adams J, Keesey J, Hicks J, DeCristofaro A (2003). The quality of health care delivered to adults in the United States. N England Journal of Medicine.

[5] Squires JE, Graham ID, Grinspun D, Lavis J, Legare F, Bell R, et al. Inappropriateness of health care in Canada: a systematic review protocol. Systematic Review. 2019;8(50). 10.1186/s13643-019-0948-1. 10.1186/s13643-019-0948-1 PMC6371550 30744703

[6] Innis J, Berta W. Routines for change: How managers can use absorptive capacity to adopt and implement evidence-based practice. Journal of Nursing Management. 2016;24(6). doi:10.1111/jonm.12368. 10.1111/jonm.12368 26893195

[7] Bornbaum C, Kornas K, Pierson L, Rosella LC. Exploring the function and effectiveness of knowledge brokers as facilitators of knowledge translation in health-realted settings: a systematic review and thematic analysis. Implement Sci. 2015;10. 10.1186/s13012-015-0351-9. 10.1186/s13012-015-0351-9 PMC4653833 26589972

[8] Currie G, Burgess N, White L, Lockett A, Gladman J, Waring J. A qualitative study of the knowledge-brokering role of middle-level managers in service innovation: managing the translation gap in patient safety for older persons’ care. Health Services and Delivery Research. 2014;2(32). 10.3310/hsdr02320. 25642513

[9] Canadian Health Services Research Foundation. The theory and practice of knowledge brokering in Canada’s health system. 2003. Ottawa, Ontario: www.chsrf.ca.

[10] Flodgren G, Parmelli E, Doumit G, Gattellari M, O’Brien MA, Grimshaw J, et al. Local opinion leaders: effects on professional practice and health care outcomes. Cochrane Database Systematic Review. 2011;8. 10.1002/14651858.CD00125. 10.1002/14651858.CD000125.pub4 PMC4172331 21833939

[11] Soo S, Berta W, Baker G (2009). Role of champions in the implementation of patient safety practice change. Healthcare Quarterly.

[12] Thompson GN, Estabrooks CA, Degner LF (2006). Clarifying the concepts in knowledge transfer: a literature review. J Adv Nurs.

[13] Glegg S (2010). Knowledge brokering as an intervention in paediatric rehabilitation practice. Int J Ther Rehabil.

[14] Rivard LM, Russell DJ, Roxborough L, Ketelaar M, Bartlett DJ, Rosenbaum P (2010). Promoting the use of measurement tools in practice: a mixed methods study of the activities and experiences of physical therapist knowledge brokers. Phys Ther.

[15] Russell D, Rivard LM, Walter SD, Rosebaum PL, Roxborough L, Cameron D, et al. Using knowledge brokers to facilitate the uptake of pediatric measurement tools into clinical practice: a before-after intervention study. Implementation Science. 2010;5(92). 10.1186/1748-5908-5-92 PMC3004810 21092283

[16] Dobbins M, Traynor RL, Workentine S, Yousefi-Nooraie R, Yost J (2018). Impact of an organization-wide knowledge translation strategy to support evidence- informed public health decision making. BMC Public Health.

[17] Dobbins M, Greco L, Yost J, Traynor R, Decorby-Watson K, et al. A description of a tailored knowledge translation intervention delivered by knowledge brokers within public health departments in Canada. Health Research Policy and Systems. 2019;17(63):1–8. 10.1186/s12961-019-0460-z. 10.1186/s12961-019-0460-z PMC6585045 31221187

[18] Birkin S, Clary A, Tabriz AA, Turner K, Meza R, Zizzi A, et al. Middle managers’ role in implementing evidence-based practices in healthcare: a systematic review. Implementation Science. 2018;13(149): 1-14. Available from: 10.1186/s13012-018-0843-5. 10.1186/s13012-018-0843-5 PMC6292008 30541596

[19] Urquhart R, Kendell C, Folkes A, Reiman T, Grunfeld E, Porter GA (2019). Factors influencing middle mangers’ commitment to the implementation of innovation in cancer care. J Health Services Res Pol.

[20] Burgess N, Currie G (2013). The knowledge brokering role of the hybrid middle level manager: the case of healthcare. Br J Manag.

[21] Van Eerd D, Newman K, DeForge R, Urquhart R, Cornelissen E, Dainty KN (2016). Knowledge brokering for healthy aging: a scoping review of potential approaches. Implement Sci.

[22] Glegg SM, Hoens A (2016). Role domains of knowledge brokering: a model for the health care setting. JNPT..

[23] Schleifer Taylor J, Verrier MC, Landry MD (2014). What do we know about knowledge brokers in pediatric rehabilitation? A systematic search and narrative summary. Physiother Can.

[24] Ward V, Hamer S, House A (2009). Knowledge brokering: exploring the process of transferring knowledge into action. BMC Health Serv Res.

[25] Cranley LA, Cummings GG, Profetto-McGrath J, Toth F, Estabrooks CA. Facilitation roles and characteristics associated with research use by healthcare professionals: a scoping review. BMJ Open. 2016;7. 10.1136/bmjopen-2016-014384. 10.1136/bmjopen-2016-014384 PMC5724142 28801388

[26] Dixon-Woods M, Cavers D, Agarwal S, Annandale E, Arthur A, Harvey J (2006). Conducting a critical interpretive synthesis of the literature on access to healthcare by vulnerable groups. BMC Med Res Methodol.

[27] Kangasniemi M, Kallio H, Pietila AM (2013). Towards environmentally responsible nursing: a critical interpretive synthesis. J Adv Nurs.

[28] Tong A, Flemming K, McInnes E, Oliver S, Craig J. Enhancing transparency in reporting the synthesis of qualitative research: ENTREQ. BMC Medical Research Methodology. 2012;12(181) Available from: http://www.biomedcentral.com/1471-2288/12/181. 10.1186/1471-2288-12-181 PMC3552766 23185978

[29] Shenton K (2004). Strategies for ensuring trustworthiness in qualitative research projects. Educ Inf.

[30] McGowan J, Sampson M, Lefebvre C (2010). An evidence based checklist for the peer review of electronic search strategies (PRESS EBC). Evid Based Libr Inf Pract.

[31] Flemming K (2010). Synthesis of quantitiative and qualitative research: an example of critical interpretive synthesis. J Adv Nurs.

[32] Standards for Reporting Qualitative Research. (2017, April 15). Available from: http://www.equator-network.org.

[33] O'Cathain A, Murphy E, Nicholl J (2008). The quality of mixed methods studies in health services research. J Health Serv Res Policy.

[34] Roever L. Critical appraisal of a questionnaire study. Evidence Based Medicine and Practice. 2016;1(2). Available from: 10.4172/ebmp.1000e110.

[35] Ogrinc G, Davies L, Goodman D, Batalden P, Davidoff F, Stevens D. SQUIRE 2.0 (Standards for Quality Improvement Reporting Excellence): revided publication guidelines from a detailed consensus process. The Journal of Continuing Education in Nursing. 2015; 46(11):501–507. Available from: 10.3928/00220124-20151020-02. 10.3928/00220124-20151020-02 26509402

[36] Tufanaru C, Munn Z, Aromataris E, Campbell J, Hoop L. Systematic reviews of effectiveness. In: Aromataris E, Munn Z, editors. Joanna Briggs Institute Reviewers Manual. The Joanna Briggs Institute. 2017. Available from: https://reviewersmanual.joannabriggs.org.

[37] Birkin SA, DiMartino LD, Kirk MA, Lee S, McCelland M, Albert NA (2016). Elaborating on theory with middle managers’ experience implementing healthcare innovations in practice. Implement Sci.

[38] Johnson M, Tod A, Collins K, Brummell S (2015). Prognostic communication in cancer: a critical interpretive synthesis of the literature. Eur J Oncol Nurs.

[39] Bullock A, Morris ZS, Atwell C (2012). Collaboration between health services managers and researchers: making a difference. Original Research.

[40] Donahue KE, Halladay JR, Wise A, Reiter K, Lee SD, Ward K (2013). Facilitator of transforming primary care: a look under the Hood at practice leadership. Ann Fam Med.

[41] Kakyo TA, Xiao LD (2017). Nurse managers’ experiences in continuous quality improvement in resource-poor healthcare settings. Nurs Health Sci.

[42] Kitson A, Silverton H, Wiechula R, Zeitz K, Marcoionni D, Page T (2011). Clinical nursing’, team members’ and service managers’ experiences of implementing evidence at a local level. J Nurs Manag.

[43] Schreiber J, Marchetti GF, Racicot B, Kaminski E (2015). The use of knowledge translation program to increase use of standardized outcome measures in outpatient pediatric physical therapy clinic: administrative case report. American Physical Therapy Association.

[44] Traynor R, DeCorby K, Dobbins M. Knowledge brokering in public health: a tale of two studies. The Royal Society for Public Health. 2014;128:533–544. 10.1016/j.puhe.2014.01.015. 10.1016/j.puhe.2014.01.015 24684852

[45] Catallo C (2015). Should nurses be knowledge brokers? Competencies and organizational resources to support the role. Nurs Leadersh.

[46] Engle RL, Lopez ER, Gormley KE, Chan JA, Charns MP, VanDeusen Lukas C (2017). What roles do middle managers play in implementation of innovative practices?. Health Care Manag Rev.

[47] Hitch D, Rowan S, Nicola-Richmond K. A case study of knowledge brokerage in occupational therapy. MA Healthcare Ltd. 2014.

[48] Urquhart R, Kendell C, Folkes A, Reiman T, Grunfeld E, Porter GA (2018). Making it happen: middle managers’ roles in innovation implementation in health care. Worldviews Evid-Based Nurs.

[49] Ploeg J, Skelly J, Rowan M, Edwards N, Davies B, Grinspun D. The role of nursing best practice champions in diffusing practice guidelines: a mixed methods study. Worldviews Evid-Based Nurs. 2010:238–51. 10.1111/j.1741-6787.2010.00202. 10.1111/j.1741-6787.2010.00202.x 20880009

[50] Fleiszer AR, Semenic SE, Ritchie JA, Richer MC, Denis JL (2015). Nursing unit leaders’ influence on the long-term sustainability of evidence-based practice improvements. J Nurs Manag.

[51] Jeffs L, Indar A, Harvey B, McShane J, Bookey-Bassett S, Flintoft V (2016). Enabling role of manager in engaging clinicians and staff in quality improvement. J Nurs Care Qual.

[52] Warshawsky NE, Lake SW, Bradford A (2013). Nurse managers describe their practice environment. Nurs Admin Q.

[53] Gutberg J, Berta W (2017). Understanding middle managers’ influence in implementing patient safety culture. BMC Health Serv Res.

[54] Kislov R, Hodgson D, Boarden R (2016). Professionals as knowledge brokers: the limits of Authority in Healthcare Collaboration. Public Adm.

[55] Shaw L, McDermid J, Kothari A, Lindsay R, Brake P, Page P (2010). Knowledge brokering with injured workers: perspective of injured worker groups and health care professionals. Work..

[56] Wilson L, Orff S, Gerry T, Shirley BR, Tabor D, Caiazzo K (2013). Evolution of an innovative role: the clinical nurse leader. J Nurs Manag.

[57] Currie G, Burgess NA, Hayton JC (2015). HR practices and knowledge brokering by hybrid middle managers in hospital settings: the influence of professional hierarchy. Human Resources Management.

[58] Waring J, Currie G, Crompton A, Bishop S (2013). An exploratory study of knowledge sharing and learning for patient safety?. Soc Sci Med.

[59] Girard A, Rochette A, Fillion B (2013). Knowledge translation and improving practices in neurological rehabilitation: managers’ viewpoint. Journal of Evaluation in Clinical Practice.

[60] Williams L, Burton C, Rycroft-Malone J (2012). What works: a realist evaluation case study of intermediaries in infection control practice. J Adv Nurs.

[61] Bradley EH, Holmboe ES, Mattera JA, Roumanis SA, Radford MJ, Krumholz HM (2003). The roles of senior Management in Quality Improvement Efforts: what are the key components?. J Healthc Manag.

[62] Ott KM, Haddock KS, Fox SE, Shinn JK, Walters SE, Hardin JW (2009). The clinical nurse leader: impact on practice outcomes in the veterans health administration. Nurs Econ.

[63] Schell WJ, Kuntz SW (2013). Driving change from the middle: an exploration of the complementary roles and leadership Behaviours of clinical nurse leaders and engineers in healthcare process improvement. Eng Manag J.

[64] Jeffs LP, Lo J, Beswick S, Campbell H (2013). Implementing an organization-wide quality improvement initiative. Nursing Administrative Quarterly.

[65] Lalleman PCB, Smid GAC, Lagerwey MD, Oldenhof L, Schuurmans MJ (2015). Nurse middle managers’ dispositions of habitus: a Bourdieusian analysis of supporting role Behaviours in Dutch and American hospitals. Adv Nurs Sci.

[66] Birken SA, Lee S, Weiner BJ, Chin MH, Chiu M, Schaefer CT (2015). From strategy to action: how top managers’ support increases middle managers’ commitment to innovation implementation in health care organization. Health Care Manag Rev.

[67] Bradley EH, Webster TR, Schlesinger M, Baker D, Inouye SK (2006). The roles of senior Management in Improving Hospital Experiences for frail older adults. J Healthc Manag.

[68] Balding C (2005). Embedding organisational quality improvement through middle manager ownership. International Journal of Health Care Quality Assurance.

[69] Uvhagen H, Hasson H, Hansson J, von Knorring M (2018). Leading top-down implementation processes: a qualitative study on the role of managers. BMC Health Serv Res.

[70] Fryer A-K, Tucker AL, Singer SJ (2018). The impact of middle manager affective commitment on perceived improvement program implementation success. Health Care Manag Rev.

[71] Chang Chen H, Jones MK, Russell C (2013). Exploring attitudes and barriers toward the use of evidence-based nursing among nurse managers in Taiwanese residential aged care facilities. J Gerontol Nurs.

[72] Hansell V (2018). Identifying the prevalence of influential factors on middle manager’s ability to lead organizational change within the context of community nursing and therapy services. Int J Healthcare Management.

[73] Buick F, Blackman D, Johnson S (2017). Enabling middle managers as change agents: why organizational support needs to change. Aust J Public Adm.

[74] Breed M, Downing C, Ally H. Factors influencing motivation of nurse leaders in a private hospital group in Gauteng, South Africa: A quantitative study. Curationis. 2020;43(1): Available from: 10.4102/curationis.v43i1.2011. 10.4102/curationis.v43i1.2011 PMC7059169 32129642

[75] Kallas KD (2014). Profile of an excellent nurse manager: identifying and developing health care team leaders. Nurse Admin Quarterly.

[76] Sellgren S, Ekvall G, Tomson G (2006). Leadership styles in nursing management: preferred and perceived. J Nurs Manag.

[77] Dobbins M, DeCorby K, Robeson P, Ciliska D, Hanna S, Cameron R, et al. A description of a knowledge broker role implemented as part of a randomized controlled trial evaluating three knowledge translation strategies. Implementation Science. 2009;4(23). Available from: http://www.implementationscience.com/content/4/1/23. 10.1186/1748-5908-4-23 PMC2680804 19397820

[78] Cook MJ (2001). The attributes of effective clinical nurse leaders. Nurs Stand.

[79] Gentry H, Prince-Paul M. The nurse influencer: a concept synthesis and analysis. Nurs Forum. 2020:1–7. 10.1111/nuf.12516 33145784

[80] Demes JAE, Nickerson N, Farand L, Montekio VB, Torres P, Dube JG (2020). What are the characteristics of the champion that influence the implementation of quality improvement programs?. Evaluation and Program Planning.

[81] Bonawitz K, Wetmore M, Heisler M, Dalton VK, Damschroder LJ, Forman J (2020). Champions in context: which attributes matter for change efforts in healthcare?. Implementation Sci.

[82] Bunce AE, Grub I, Davis JV, Cowburn S, Cohen D, Oakley J (2020). Lessons learned about effective operationalization of champions as an implementation strategy: results from a qualitative process evaluation of a pragmatic trial. Implementation Sci..

[83] Birkin SA, Currie G (2021). Using organization theory to position middle-level managers as agents of evidence-based practice implementation. Implementation Sci.

[84] Klein KI, Sorra JS (1996). The challenge of innovation implementation. Acad Manag Rev.

[85] Tricco AC, Antony J, Soobiah C, Kastner M, MacDonald H, Cogo E (2016). Knowledge synthesis methods for integrating qualitative and quantitative data: a scoping review reveals poor operationalization of the methodological steps. J Clin Epidemiol.

[86] Tricco AC, Antony J, Soobiah C, Kastner M, MacDonald H, Cogo E (2016). Knowledge synthesis methods for generating or refining theory: a scoping review reveals that little guidance is available. J Clin Epidemiol.

