# Supplementary material for: The roles, activities and impacts of middle managers who function as knowledge brokers to improve care delivery and outcomes in healthcare organizations: a critical interpretive synthesis
Source: BMC Health Serv Res. 2022 Jan 2;22:11. doi: 10.1186/s12913-021-07387-z (PMC8722036; doi:10.1186/s12913-021-07387-z)
Supplement: Supplementary file 1 — Additional file 1. ENTREQ checklist [file 12913_2021_7387_MOESM1_ESM.docx]

**Enhancing transparency in reporting the synthesis of qualitative research: the ENTREQ statement** [27]

| **No.** | **Item** | **Description** |
| --- | --- | --- |
| **1** | Aim | 3 compass questions used to guide synthesis. 1. What roles and activities do MMs enact in hospitals to function as knowledge brokers? 2. What are the determinants (facilitators and barriers) of MMs functioning as knowledge brokers in hospitals? 3. How do MM KBs impact hospital performance? |
| **2** | Synthesis methodology | Critical Interpretive Synthesis (CIS) was described, and rationale provided. Page 5, line 73 |
| **3** | Approach | Comprehensive and iterative search strategy provided. Page 5, line 81 |
| **4** | Inclusion criteria | Inclusion and exclusion criteria specified. Page 5, line 90 |
| **5** | Data sources | MEDLINE, CINAHL, Social Sciences Abstracts, ABI/INFORM, EMBASE, PubMed, PsycINFO, ERIC and the Cochrane Library. Page 5, line 82. |
| **6** | Electronic search strategy | Sample search strategy provided in Supplementary file 2. |
| **7** | Study screening methods | FB and ARG independently screened a sample of 50 titles and abstracts according to the eligibility criteria and compared and discussed results. Based on discrepancies, they then modified the eligibility criteria and discussed how to apply them. Thereafter, FB screened all remaining studies, and discussed all uncertainties with ARG and the research team. FB retrieved all titles and abstracts deemed “potentially relevant”. FB and ARG independently screened a sample of 25 full-text articles, and again discussed selection discrepancies to standardize how eligibility applied. Thereafter, FB screened all remaining full-text items. Page 6, line 105. |
| **8** | Study characteristics | Described in Results section of manuscript. Page 8, line 151. |
| **9** | Study selection results | The initial search yielded 9,936 articles. Following removal of duplicates, 9,760 titles were not eligible, and 176 items were retrieved as potentially relevant. Of the potential articles, 135 were excluded because the study design was ineligible (25), they did not examine MMs (27) or KBs (34), were not focused on the evaluation of an MM KB role (39), were editorials (4), or the publication was a duplicate (6) leaving 41 articles eligible for review. See Figure 1. |
| **10** | Rationale for appraisal | As per CIS, quality appraisal conducted to assess the relevance and quality of the eligible studies. |
| **11** | Appraisal process | FB and ARG independently assessed and compared the quality of a sample of seven studies each. Thereafter, FB assessed the quality of the remaining studies. |
| **12** | Appraisal items | We used quality appraisal tools relevant to different research designs: Standards for Reporting Qualitative Research (SRQR) [31], the Good Reporting of a Mixed Methods Study (GRAMMS) tool [32], Critical Appraisal of a Questionnaire Study [33], Revised Standards for Quality Improvement Reporting Excellence (SQUIRE 2.0) tool [34], and the Critical Appraisal Checklist for Quasi-Experimental Studies [35]. |
| **13** | Appraisal results | See Supplementary file 4 |
| **14** | Data extraction | See data extraction form in Supplementary file 3 |
| **15** | Software | NVivo 12 software |
| **16** | Number of reviewers | Four members of the research team |
| **17** | Coding | As per CIS, this was an iterative process where codes were assigned to significant elements of data then grouped into relevant categories and organized into preliminary themes. Page 7, line 127. |
| **18** | Study comparison | As per CIS methodology, an integrative grid was constructed to facilitate the management and analysis of the articles included in the review and to assist with coding, identifying categories and themes and the relationships among them to develop synthetic constructs. A constant comparative method was used to ensure emerging synthetic constructs were grounded in the data. Page 7, line 136. |
| **19** | Derivation of themes | Inductive |
| **20** | Quotations | See Table 2, page 33 |
| **21** | Synthesis output | A new organizing framework. Page 13, line 276 |
