# Supplementary material for: The roles, activities and impacts of middle managers who function as knowledge brokers to improve care delivery and outcomes in healthcare organizations: a critical interpretive synthesis
Source: BMC Health Serv Res. 2022 Jan 2;22:11. doi: 10.1186/s12913-021-07387-z (PMC8722036; doi:10.1186/s12913-021-07387-z)
Supplement: Supplementary file 2 — Additional file 2. Search strategy [file 12913_2021_7387_MOESM2_ESM.docx]

## Sample search strategy

Sample Search Strategy for MEDLINE
Database: Ovid MEDLINE(R) In-Process & Other Non-Indexed Citations and Ovid MEDLINE(R) <1946 to Present>

Search Strategy:

-------------------------------------------------------------------------------------------------------------------------

1 exp Leadership/ (37403)

2 exp Nurse Administrators/ (12743)

3  exp Physician Executives/ (4120)

4 exp Hospital Administrators/ (8246)

5 (director* or executive* or manager* or leader* or middle manager* or administrator* or nurse administrator* or

hospital administrator* or physician executive*).mp. [mp=title, abstract, original title, name of substance word,

subject heading word, keyword heading word, protocol supplementary concept word, rare disease supplementary concept

word, unique identifier, synonyms] (223639)

6 1 or 2 or 3 or 4 or 5 (223639)

7 exp Role/ (104331)

8 role*.mp. [mp=title, abstract, original title, name of substance word, subject heading word, keyword heading word,

protocol supplementary concept word, rare disease supplementary concept word, unique identifier, synonyms] (2589872)

9  ((knowledge or evidence or finding or information) and (broker* or intermediar* or mediator* or liaison or

navigator or facilitator or translator or leader or enabler)).mp. [mp=title, abstract, original title, name of substance

word, subject heading word, keyword heading word, protocol supplementary concept word, rare disease supplementary

concept word, unique identifier, synonyms] (40431)

10  7 or 8 or 9 (2613358)

11 exp Translational Medical Research/ (8882)

12 exp Information Dissemination/ (14744)

13 exp "Diffusion of Innovation"/ (18754)

14  ((information or research or knowledge or evidence or finding*) and (disseminat* or innovat*)).mp. [mp=title,

abstract, original title, name of substance word, subject heading word, keyword heading word, protocol supplementary

concept word, rare disease supplementary concept word, unique identifier, synonyms] (151084)

15  11 or 12 or 13 or 14 (166525)

16 exp Hospitals/ (257261)

17 (healthcare or health care).mp. [mp=title, abstract, original title, name of substance word, subject heading

word, keyword heading word, protocol supplementary concept word, rare disease supplementary concept word, unique

identifier, synonyms] (834259)

18 16 or 17 (1038937)

19 performance or quality improvement or patient safety or continuous improvement or quality care).mp. [mp=title,

abstract, original title, name of substance word, subject heading word, keyword heading word, protocol supplementary

concept word, rare disease supplementary concept word, unique identifier, synonyms] (898111)

20 "Outcome Assessment (Health Care)"/ (66459)

21 exp Patient Outcome Assessment/ (4893)

22 exp Treatment Outcome/ (905701)

23 19 or 20 or 21 or 22 (1827937)

24 6 and 10 and 15 and 18 and 23 (405)

25 limit 24 to (english language and yr="2001 -Current") (361)
