# Supplementary material for: The roles, activities and impacts of middle managers who function as knowledge brokers to improve care delivery and outcomes in healthcare organizations: a critical interpretive synthesis
Source: BMC Health Serv Res. 2022 Jan 2;22:11. doi: 10.1186/s12913-021-07387-z (PMC8722036; doi:10.1186/s12913-021-07387-z)
Supplement: Supplementary file 3 — Additional file 3. Eligibility criteria [file 12913_2021_7387_MOESM3_ESM.docx]

**Eligibility Criteria based on the PICO Framework**

| **Eligible** |  |
| --- | --- |
| **Population**  **/Participants** | Peer reviewed studies of:   - Middle Managers as Knowledge Brokers, Middle Managers, Knowledge Brokers - specific Knowledge Broker and Middle Manager roles, activities, tasks - specific Middle Manager as Knowledge Broker roles, activities, tasks - Context is health care: hospitals or other health related settings; may need to broaden to management and business literature - Focus Middle Manager(s) but broaden to Managers, hospital administrators, nurse administrators, directors, physician executives, operational leaders |
| **Interventions** | - Studies of how Knowledge Brokers operate in practice - for the creation, use and sharing of knowledge, for implementation of evidence-based practice(s) - Studies of how Middle Managers operate in practice – for the creation, use and sharing of knowledge, for implementation of evidence-based practice(s), for innovation implementation - Evaluation of Knowledge Brokers, Middle Managers, Middle Managers as Knowledge Brokers - Experiences, perspectives, attitudes of Knowledge Brokers, Middle Managers as Knowledge Brokers - Training of Knowledge Brokers, Middle Managers, or Middle Managers as Knowledge Brokers |
| **Comparison/**  **Publication Type** | Published studies that explore or describe:   - Knowledge Brokers, Middle Managers, Middle Managers as Knowledge Brokers’ attributes, roles, approaches, activities, tasks, domains - Determinants (challenges/barriers or facilitators/enablers) of Knowledge Brokers or Middle Managers as Knowledge Brokers (perceived or experienced) - Impact of Knowledge Brokers, Middle Managers as Knowledge Brokers on care delivery/patient outcomes - Studies that compare Knowledge Brokers, or Middle Managers as Knowledge Brokers across sites   Publication Types:   - Quantitative – randomized or pragmatic controlled trials, case studies, questionnaire surveys - Mixed-Methods studies that describe use of Knowledge Brokers, Middle Managers, Middle Managers as Knowledge Brokers - Qualitative – interviews, focus groups, qualitative single or multiple case studies - Theoretical or conceptual papers focused on Knowledge Brokers, MMs, or Middle Managers as Knowledge Brokers |
| **Outcomes** | - Any outcomes reported by eligible studies - Knowledge Broker effectiveness (change in knowledge, skills, policies and/or practices, care delivery, satisfaction in role) - Middle Managers as Knowledge Brokers outcomes (change in knowledge, skills, policies and/or practices, satisfaction in role) - Barriers and facilitators of Knowledge Brokering, Middle Managers as Knowledge Brokers - Descriptions of Knowledge Brokers, Middle Managers, Middle Managers as Knowledge Brokers - Studies to educate/train Knowledge Brokers, MMs, Middle Managers as Knowledge Brokers - Pilot testing of Knowledge Broker role, Middle Manager, and MM as Knowledge Broker role |
| **Not Eligible** |  |
|  | - Non-English articles - Grey Literature - Non-health care management roles, patients, consumers - Systematic reviews (will check reference lists for eligible studies) - Protocols - Abstracts, editorials, letters, unpublished theses, conference proceedings, commentaries |
