# Supplementary material for: The roles, activities and impacts of middle managers who function as knowledge brokers to improve care delivery and outcomes in healthcare organizations: a critical interpretive synthesis
Source: BMC Health Serv Res. 2022 Jan 2;22:11. doi: 10.1186/s12913-021-07387-z (PMC8722036; doi:10.1186/s12913-021-07387-z)
Supplement: Supplementary file 4 — Additional file 4. Data extraction form for eligible studies [file 12913_2021_7387_MOESM4_ESM.docx]

Data extraction form of eligible studies

| **First Author & Year** | **Journal** | **Country** | **Purpose** | **Label for MM/KB definition** | **Research design** | **Participants** | **Intervention** | **Impact/Findings** | **Leader characteristics** | **Roles/Activities** | **Enablers** | **Barriers** |
| --- | --- | --- | --- | --- | --- | --- | --- | --- | --- | --- | --- | --- |
| Balding 2005  [60] | Int J Health Care QA | Australia | To strengthen the MM role in hospital QI program | MM defined as department heads, often a clinician (Nurse unit managers and allied health practitioners, the majority mixed a clinical and managerial role, responsible for running a clinical service department | Case study, quasi-experimental | N=35 MM, nursing & allied health & some non-clinical | Development and evaluation of a MM driven QI model implemented in one hospital over 12 months. MM attitude to org culture, their role & QI program surveyed by questionnaire pre- and post- model development | MM understanding & ownership of QI program & implementation significantly increased, although perceived enjoyment of being involved in QI (pride in org, showing initiative & communication) decreased. QI Model has 5 elements:   1. Senior management commitment & leadership, 2. Education and information, 3. Senior and MM QI role accountability, 4. MM involvement in QI planning, 5. MM own & operate the QI program |  | - Providing safe and effective care - Contributing to patients achieving positive outcomes - Running an efficient and effective service - Ensuring the patient’s encounter with my department is positive - Agreement in MM’s perception of their role: - Participate in decisions relevant to my role - Opportunity to use a range of skills in my role - Participation in QI a professional obligation and an important part of their role | - More time in the day - More resources - Education and training in QI - More evidence of improvements - Support from senior management - Experience in QI | Insufficient time and resources |
| Birkin 2015  [47] | Health Care Mgmt Review | USA | To assess the ways in which top manager's support influences MMs commitment to innovation implementation | Top Managers: chief executive officers, medical directors | Mixed methods sequential design | 120 MMs surveyed and 16 interviewed |  | Results suggest that top managers increase middle managers’ commitment by directly conveying to middle managers that innovation implementation is an organizational priority (" = 0.37, p = .09); allocating implementation policies and practices including performance reviews, human resources, training, and funding (bootstrapped estimate for performance reviews = 0.09; 95% confidence interval [0.03, 0.17]); and encouraging middle managers to leverage performance reviews and human resources to achieve innovation implementation. |  | NR | NR |  |
| Birkin 2016  [64] | Implementation Science | USA | To elaborate on MM role theory & assess alignment with MM experience | MM: employees who are supervised by an organization's top managers and who supervise frontline employees | Survey | 63 MMs | Self-administered questionnaire distributed at a nursing summit | MMs experience aligned well with the theory and expanded definitions of the roles and activities hypothesized. Ranked all 4 hypothesized roles as extremely important but ranked diffusing and synthesizing most important and selling innovation as least important. |  | MMs influence implementation effectiveness by fulfilling four roles:   1. Obtaining and diffusing information: MMs can provide frontline staff with information necessary to implement innovations, field questions from staff, and disseminate information regarding material and emotional support for the implementation 2. Adapting information and innovation: MMs can use daily conversations to help frontline staff understand key information regarding the innovation, monitor staff responses to the information, reinterpret and make this information relevant to the staff and the organizational context 3. Mediating between strategy and day-to-day activities: MMs can provide staff with the tools necessary to implement innovations, measure performance and engage in frontline activities 4. Selling innovation implementation: MMs can justify the innovation implementation and encourage staff to consistently and effectively use those innovations |  |  |
| Bradley 2006  [55] | J Healthcare Mgmt | USA | Understand senior manager roles in implementation of evidence - based Hospital Elder Life Program of 6 interventions that reduce risk of delirium among older hospitalized adults | Administrative champions included: chief operating officers, vice presidents, medical directors, chief nursing officers, director of volunteers, or program directors  An individual in a high-level position with substantial administrative responsibility | Mixed methods: web survey of 63 hospitals and 102 qualitative interviews every 6 months for 2 years with staff from 13 hospitals | 63 hospitals in web-based survey, 13 hospitals in staff interviews |  | Number of hospitals that implemented HELP interventions: Orientation and therapeutic activities (100%), aiding sleep (79.3%), early mobilization (96.6%), visual aids and reminders (96.4%), hearing aids and reminders (96.5%), dehydration protocol (93.1%)  Perceived outcomes were:   - Reduced length of stay - Reduced cost per patients - Decreased episodes of delirium - Reduced Falls - Reduced use of restraints - Reduced use of sitters - Reduced discharges to nursing homes - Reduced functional decline - Increased patient satisfaction - Increased nurses’ job satisfaction - Increased nurse retention - Increased nurses’ knowledge - Increased physicians’ job satisfaction - Reduced incontinence Increased nurses’ knowledge - Reduced pressure ulcers - Reduced unscheduled readmissions - Increased nurses’ knowledge - Increased nurse aides’ knowledge - Increased physicians’ knowledge - Increased residents’ knowledge - Increased pharmacists’ knowledge - Increased therapists’ knowledge |  | Hospital managers advocated for the program in a variety of ways including:   - Providing resources for needed staffing or staff training; - Promoted the program among physicians who were initially less involved, other administrators and the governing board | Senior management support   - Consistency with hospital mission - Commitment of nurse leader - Commitment of physician leader - Evidence of positive patient outcomes - Hospital financial support - Evidence of cost-effective-ness | - Lack senior management support - Too expensive - No nurse leader - No physician leaders - Lack of support from nurses or physicians |
| Bradley 2003  [53] | J Healthcare Mgmt | USA | 1. To define the key roles and activities of senior managers in QI 2. Develop a taxonomy to classify these roles and activities 3. Propose key elements of management involvement most central to successful QI efforts directed at physician prescription of beta blockers after acute MI | Senior Managers not defined | Qualitative interviews | 8 hospitals that represented a range of improvement rates in beta blocker rates with 45 participants: 14 medical staff, 15 nursing staff, 11 quality management staff, and 5 senior managers |  | Participants’ descriptions of the nature and level of management involvement and support for QI efforts differed substantially among the hospitals. In higher performing hospitals, senior managers were personally engaged in advocating for QI in AMI care, had good working relationships with medical staff, supported norms of team collaboration & ensured availability of resources. In lower performing hospitals, senior managers did not participate on teams and were not viewed as advocating for QI in AMI care, had poor relationships with medical staff and lacked resources to collect and disseminate data to improve care. |  | 5 common roles & activities of senior manager involvement in and support of QI:   - Personal engagement - Relationship with clinical staff - Promotion of an organizational culture - Support of QI with organizational structures - Procurement of organizational resources |  |  |
| Bullock 2012 [56] | J Health Serv Res Policy | UK | To evaluate if the involvement of healthcare managers in research projects improves the quality & relevance of research & if collaboration builds capacity in managers | Not defined | Program Evaluation - using semi-structured face to face interviews | Interviews with 10 manager fellows, chief investigators |  | Found that management fellows (MF) did have an impact on the quality and relevance of research, and manager fellows did develop the capacity to make use of research. | MFs personal characteristics contributed to success:   - Willingness to learn and contribute to research - Engage and be proactive in disseminating - Ongoing connection with workplace and professional colleagues to exchange knowledge and insights | - Engaged key informants and knew gatekeepers to assist in recruitment - Used clinical expertise to help research team make feasible and ethical approaches to patients - Able to open doors because of their networks – knew the right people - Used contextual understanding to improve the design of data collection tools and processes - Contributed to the design of data collection processes to enhance response rates - Piloted the recruitment process and interview schedule - Provided a reality check for researchers’ analysis - Supported and offered guidance on disseminating activities | - Know-ledge and experience of the NHS - Characteristics of the fellows - Had an operational or strategic role - Mutual respect and being valued- reported feeling supported and valued by researchers - Timing and flexibility | - When Manager fellows did not have the networks or skills - Lack of clarity about the purpose of the program - Costs to the fellow - Timing and flexibility |
| Catallo 2015 [65] | Nursing Leadership | Canada | To examine the competencies that Nurse Knowledge Brokers should possess & attributes associated with the role. | Nurse Knowledge Broker (NKB) - registered nurses with graduate preparation, extensive clinical experience and ability to be a credible and respected resource by their peers | theoretical/ conceptual paper |  | NR | ADD | NKB attributes:   - Graduate level education with exposure to research methods - Minimum 5 years of clinical experience in clinical area where KB will work | NKB competencies:   - Education and experience with EIDM process - Established networking skills to bring researchers, decision-makers, stakeholders & policy makers together - Experience with large scale implementation of change such as BPG across a department or organization - Capacity development- experience developing capacity among staff to develop a new skill or innovation & facilitation skills for presentations, meetings, workshops - Fit with organization - Accountability – credible and respected internally and externally | Organizational resources to support KB role:   - KB job description - Ongoing education-al opportunities in EIDM methods - Organizational structure and culture to support EIDM - Protected time to carry out KB functions - Mentor-ship opportunities with other experienced KBs - Resources such as access to library services - Formalized networking opportunities - Support from nurse leader to make linkages with influential leaders within the organization & external stakeholders | NR |
| Chang 2013 [69] | J of Gerontological Nsg | Taiwan | To understand the attitudes and barriers toward EBP among nurse managers | Not defined | Qualitative – semi structured interviews | N=6 nurse managers in 6 residential aged care facilities | NR | Most expressed positive attitudes toward research and EBP but reported little experience in its implementation. Barriers were identified and implications discussed |  |  |  | - Lack of motivation & confidence - Difficulty finding and understanding research articles - Lack of funding, time - Lack of authority - Workplace culture |
| Currie 2015 [40] | Human Resource Mgmt | UK | To understand the interaction between HR practices & Knowledge brokering by Hybrid MMs | Hybrid middle managers are defined as mediating persons capable of working through sets of ideas belonging to management and those belonging to clinical practice | Qualitative -3 sequential phases:  Phase 1&2 used semi-structured interviews,  Phase 3 combined semi-structured interviews, focus groups & observations of risk management committee following a serious incident | Phase 1: 17 executive managers, policy actors & senior academic researchers.  Phase 2: 35 hybrid Nursing MMs & 19 Hybrid Medical MMs across 3 hospitals. | NR | Suggest that: professional power and status has implications for HR practices to support knowledge brokering by HMMs. Hybrid Nurse MMs broker knowledge downward through professional hierarchy within their peer group but find it hard to broker knowledge upward. Because they lack legitimacy with doctors, Nurse MMs lack the opportunity to broker knowledge interprofessionally. Hybrid medical MMs can broker knowledge within their peer group, but higher status medical MMs may lack motivation to engage in knowledge brokering with peers outside their specialty.  Four themes identified:   1. Intraprofessional hierarchy within nursing 2. Interprofessional hierarchy between hybrid nurse MMs and doctors 3. Intraprofessional hierarchy within medicine 4. Creating opportunities for Knowledge Brokering through HR practices | NR | Nurse MMs:   - Conducted patient safety rounds - Created opportunities for junior staff to broker knowledge upwards by being visible on the wards and talking about patient safety - Set up frameworks so that effective safe care developed and maintained - Facilitated education and training of staff around quality - Focused on target setting and engaging staff in developing and meeting targets. | - Legitimizing the topic and prioritizing it for all members of the team - Linking performance management with organizational strategic priorities - Using social capital to develop relationships - Nurse MMs well prepared through experience to transition into managerial role - Purposeful cultivation of relationships between Nurse MMs and physicians - Putting people together to see the broader picture | - Physicians not prepared for the transition into managerial roles - Accountabilities have to be enforced to ensure procedures are followed - Professional status of physician specialists - did not see their role in the process - Hierarchical structure disengaged and dis-empowered Nurse MMs from physicians - Nurse MMs overburdened and time constrained - Fewer structures and processes designed to resolve the gap in patient safety knowledge within medical ranks |
| Currie 2014 [8] | Health Services and Delivery Research | UK | To generate insight into processes & structures for brokering of patient safety knowledge (PSK) by Hybrid-Middle Managers (H-MMs) in hospitals | H-MLMs: hold responsibility for clinical service delivery as well as a manager role | Mixed methods: interviews, social network analysis, observation, documentary analysis, tracer studies and focus groups | 127 H-MLMs, senior managers & professionals in 3 hospitals & external producers of PSK |  | Attempts by H-MLMs to broker PSK downwards or upwards are framed by policy directives& professional/managerial hierarchy. External performance targets & incentives compel H-MLMs in clinical governance to focus upon compliance & diverts attention from pulling knowledge downwards or upwards for service improvement. Lower-status H-MLMs, closer to service delivery, struggle to push endogenous knowledge upwards, because they lack professional and managerial legitimacy. There is a difference between how PSK is brokered within ranks of nurses and doctors, due to differences in hierarchal characteristics. |  |  | - Hierarchical characteristics - Professional and managerial legitimacy |  |
| Dobbins 2018 [16] | BMC Public Health | Canada | To evaluate the impact of organizational KT strategy to support evidence-informed public health decision making | KBs- facilitate the process of moving evidence into action; act as bridge between research producers and research users, promoting collaboration, interaction, & supporting the application of evidence in decision making | Case Study  3 cases from 3 public health depts. | Public Health professionals from 3 public health depts.  N= 606 staff at baseline and N= 804 at follow-up | KB provided training workshops & mentored small groups through a 7-step process of evidence-informed decision making over 22 months | Statistically significant increases in knowledge and skills were observed in all 3 depts. An increase in evidence-informed decision-making behavior was observed in staff that were intensively involved/engaged in the intervention |  |  | Organizational factors: -Strategic priority  -Leadership  -Readiness for change  -Choice of staff to receive training from KB – opinion leaders & use of train-the-trainer |  |
| Dobbins 2019 [17] | Health Research Policy & Systems | Canada | To provide a description of tailored KT intervention implemented by KBs, and reflections on the factors that facilitated or hindered implementation | Same as above | Case study – 3 cases from 3 public health depts. | Front line public health practitioners, managers, directors, and Medical Officers of Health | KBs spent 2 days/week delivering KT intervention in each case | Key factors at the organizational, team and individual level perceived by the KBs as either facilitating or hindering implementation of KT intervention & EIDM |  |  | Organization:  Strong leadership support/Champions  Library & onsite librarian  Committed financial and human resources  EIDM processes & tools  Regular contact between KB, staff, team, SM  Team:  Skills in finding and using research  Individual: curiosity, enthusiasm for culture change  Previous training & experience with EIDM | Organization:  Limited engagement of SM  Lack of infrastructure, resources, champions,  Team:  Managers who lack understanding of and time to develop EIDM skills  difficult to engage staff when EIDM mandated  Individual:  Resistance to EIDM/change in status quo.  Lack of confidence.  Competing priorities  Lack of time |
| Donahue 2013 [50] | Ann Fam Med | USA | Identify leadership characteristics and processes that improve quality of care in diabetes and asthma care | MM a nurse or nurse practitioner who interacted daily with both the lead physician/top manager and the clinical and front office staff | Mixed methods (chart review of clinical procedures; focus groups with staff, managers and clinicians) | 76 primary care practices (focus groups conducted in 12 practices) | Improving Performance in Practice (IPPI) - statewide initiative to improve primary care through coaching, reporting and quarterly collaborative meetings | Between 50% and 78% improved in the first year for diabetes measures (hemoglobin, LDL cholesterol, blood pressure, annual eye examination, nephropathy screening); between 68% and 76% of practices improved in asthma measures (severity assessed, annual flu vaccine, and a bundled measure | Leadership as judged by the IPPI coach was significantly associated with one clinical measure - patients having nephropathy screening (OR 1.37, 95% CI 1.08 to 1.74), particularly for those with higher leadership scores (OR 1.92 to 6.78). Leadership related themes: someone with a vision about the importance of the work, a middle manager who implemented the vision, and a team who believed in and were engaged in the work. | Top level leaders provided the vision: they saw the importance of QI, making systematic changes in care delivery, engaging the assistance of practice coaches in program implementation, and using population-level patient data to gauge improvements. Many also took a strategic view, recognizing opportunities for increased reimbursement in the future. Notably, in practices with greater improvement, implementation of the vision was led by someone other than the lead physician or top manager who, instead, delegated the operational authority to a middle manager, who was frequently a nurse or nurse practitioner who interacted daily with both the lead physician or top manager and the clinical and front office staff. | Teams of staff were engaged in transformation efforts, were comfortable with change and cross-training, and were accountable for explicit roles on the team. |  |
| Engle 2017 [51] | Health Care Mgmt Review | USA | To understand the roles of MMs in implementing innovative practices using Birkin et al.’s theory and the Org. Trans. Model | MM – situated between senior leaders and frontline staff in the organization and can bridge or create information gaps that may influence innovation implementation in positive or negative ways | Qualitative – analyzed interviews | N=98 staff; senior leaders to frontline staff |  | 14 themes emerged that identified promising strategies to help MMs implement innovations, and increases understanding of the role or organizational context in innovation implementation. Found that MMs influence most effective when the roles build on each other. |  | (Info Diffusion)  Communicate- clear, direct, transparent; use electronic tools and visual data to convey info to staff  (Info Synthesis)  Analyze where improvement broke down, identify root cause, seek ideas from staff;  (Mediating/strategy/day-to-day) Tie training to specific projects, coach staff, address barriers  (sell implement)  Serve as facilitator/champion for QI , use monthly meetings to update staff, review goals of training/QI | NR | NR |
| Fleiszer 2015 [41] | J of Nursing Manage-ment | Canada | To describe how actions of nursing unit leaders influenced the long-term sustainability of a best practice guideline (BPG) program on 4 acute care units. | Not reported | Qualitative descriptive case study | 39 key informants: 14 organizational & 25 subcases from 4 units (nurse managers, assistant nurse managers, educators, CNSs, nurses) |  | Higher levels of BPG sustainability occurred on units where formal leadership teams used an integrated set of strategies & activities. Two key manager strategies were: maintaining priorities & reinforcing expectations. Unit leaders may influence practice improvement sustainability by aligning vision, strategies & activities |  | Leaders from high performing units used specific activities to sustain BPGs:   1. Extending initial implementation – focused on the rationale rather than technical tasks 2. Educating and training- teaching content based on needs around BPG hot topics 3. Using reminders- used frequent verbal and visual reminders such as a census board 4. Communicating and discussing- manager’s frequent attendance at shift handover report 5. Evaluating performance and improving quality- involved bedside nurses as auditors to increase awareness and accountability for unit performance 6. Integrating changes into other initiatives – nesting BPGs within other existing or new initiatives | NR | NR |
| Fryer 2018 [67] | Health Care Mgmt Review | USA | To assess how MM affective commitment is related to their perceptions of implementation success | MM= directly supervise the work of frontline employees and who themselves report to a manager at least one hierarchical layer below the CEO | Exploratory cross-sectional survey | N= 67 nurse managers |  | MM affective commitment to a fall reduction program is positively associated with their perception of implementation success. Found 5 organizational variables associated with MM commitment. |  |  | Organizational variables:  Having: a clear implementation plan; adequate financial and personnel resources; SM support to overcome challenges  Frontline staff support | NR |
| Girard 2013 [61] | J of Eval Clin Prac | Canada | To explore the importance of knowledge transfer and improving practices from the viewpoint of managers of neurological rehabilitation programs | - Acting as managers but could hold title of program coordinator or leader - Responsibilities related to coordinating clinicians working in this field - At least one year of management experience | exploratory qualitative case study using semi-structured one-on-one interviews | N= 3 manager case studies |  | The study points to the lack of organizational structure fostering consistent KT across all clinicians. The managers believed KT was important, but it appeared to be less of a priority because of their numerous other responsibilities. Managers saw themselves as having a role in the first steps of knowledge identification & interventions in the KTA process cycle but less of a role in adapting knowledge & assessing barriers. They seem to have little or no role in last steps in the cycle, monitoring knowledge use, evaluating outcomes & sustaining knowledge use, although in an ideal world they would like to have a role. |  | - Coach –help in thinking about staff training needs - Facilitator –provide financial, physical and organizational resources - Motivator- encourage staff to keep practice up to date - Organizer- organize meetings to identify KT activities - Guide – provide guidance in adapting knowledge - Ambassador – make influential people aware of KT issues - Being creative in finding ways to overcome barriers to KT - Delegating competent individuals, the management of certain KT projects and offering support in terms of resources | NR | - Numerous responsibilities - Budget constraints |
| Glegg 2016 [18] | J of Neuro Phys Therapy | Canada | Objectives:   1. To describe the context for knowledge brokering in health care, 2. To provide an overview of KT theories applied to knowledge brokering 3. To propose a model outlining the role domains assumed in KB |  | Theoretical | NR | NR | This model can be used to inform the practice of knowledge brokering as well as professional development & evaluation strategies. It may be used to inform theory-driven research examining the effectiveness of knowledge brokering on knowledge generation & translation outcomes in health care field as well as on patient health outcomes. Role Model for Knowledge Brokering is composed of 5 role domains: information manager, linking agent, capacity builder, facilitator, and evaluator. |  | 5 roles: information manager, linking agent, capacity builder, facilitator and evaluator and offers a way of thinking about knowledge brokering as overlapping roles. |  |  |
| Gutberg & Berta 2017 [70] | BMC Health Services Research | Canada | Offers a conceptual model based on organizational learning theory to understand the influential role of MM in influencing organizational change | NR | Theoretical | NR | NR | Offer a series of propositions for MMs contribution to radical organizational change. Model presents a new way to understand the influential role of MM in influencing organizational change. Suggests that through an ambidextrous approach to org change strategy, MMs can leverage their unique position in the organization to effectively influence patient safety change. |  |  | Since middle managers are uniquely able to acquire and communicate knowledge across the organization, they can impact both strategic and operational outcomes. |  |
| Hitch 2014 [39] | Int J Therapy & Rehabilitation | Australia | To explore the process of knowledge brokering in an occupational therapy department in a regional health service | KB - intended for research related activities | Qualitative phenomenological case study | 7 partici-pants: - academic, managerial, clinical and KB position asked to reflect on pilot of a KB role introduced in OT Dept. | A part time 0.5 FTE KB role was introduced into the OT Dept. of a health service in a regional city for 6 months. | Outcomes for practice: Improved knowledge, skills, confidence & motivation were found for both the health service & academic participants. In this study, knowledge brokering was conceived as a mutual process, with all sides both contributing and benefiting in an equitable manner.  Four themes:   1. Doing research 2. Lived experience, 3. Building/cementing relationships 4. Enacting knowledge brokering |  | Knowledge Broker:   - Completed the ethics application - Played a capacity building role – introducing the process to clinicians - Prepared documents and checklists - Held face to face meetings to revise the processes and outcome measures - Gave administrative support - Applied for grants and other funding - Formed relationships with those involved in project - Involved in linking with individuals and groups | Mutual benefit & respect | - Resources - Protected time - Lack of time for clinicians to do data collection |
| Jeffs 2013  [46] | Nurse Admin Q | Canada | To explore perceptions & experiences of front-line nurses, project leads, and managers associated with an organization-wide CUE-QI initiative | Not defined | Qualitative with focus groups and interviews | Focus groups with 13 frontline nurses and interviews with 10 managers & 6 project leads | Utilizing evidence Quality Improvement (CUE-QI) initiative. Front-line nurses from a variety of clinical units focused on a specific QI initiative aimed at: improving work environments, contributing to engagement & retention of nurses, and supporting quality patient care. Took part in interactive learning modules in face to face classroom and working group sessions. Performed a rapid (6 month) test of change and monitored results, was paired with a mentor and shared their progress in a monthly CoP | Findings show the value of Communities of Practice (CoP) and ongoing mentorship for nurses as key strategies to acquire and apply QI knowledge to a QI project on their respective units.  3 key themes:   1. Improving care in a networked approach 2. Driving QI and having a sense of pride 3. Overcoming challenges |  | Managers   - Helped to ensure the feasibility of the project & reinforce the initiative on their local units - Communicated and supported the initiative | - Support from each other - Project mentor and managers - Using a feedback loop - Leveraging unit support | - Workload and time constraints - Unit culture that is unsupportive - Resistant to change from staff - Lack of support from nurses on the clinical units |
| Jeffs 2016  [42] | J Nurs Care Q | Canada | To explore the influence of managers in engaging clinicians in QI initiatives | Unit Manager not defined | Qualitative with interviews and focus groups | 47 Participants = 18 project leads, 20 nurses, 5 managers, 4 mentors participated in focus groups or an interview | QI intervention PERFORM KT was to enhance competencies for clinicians and staff to select, interpret, and use performance data to drive QI efforts at the local level. Clinicians and managers participated in an interactive learning strategy over 9 months that included face to face education modules in a monthly CoP. Participating teams selected a unit-specific QI topic and applied tools learned to evaluate their respective local performance data. | 2 themes emerged from the data:   1. Balancing being present while letting front-line staff lead QI project 2. Leveraging flexibility in scheduling with protected time. |  | - Aware of the project and checked in with staff - Provided feedback on team’s project charter - Gave updates to unit staff - Gave presentations to the larger PERFORM group - Organized and coached project leads - Ensured flexibility of nursing staff’s schedule, particularly the QI project leads - Linked them to resources that could facilitate the local QI project - Engaged other unit staff in QI efforts - Attended monthly CoPs - Set up weekly meetings with project team - Accessed funds for release time - Leveraged additional time beyond the funds - Had ongoing conversations with the project leads, clinicians, and staff ensured that “everyone was on board” for the local QI efforts | - Manager’s ongoing support - Keeping an eye on the project and staff - Making sure everyone was onboard - Acknowledging the project was ongoing and they were aware of it - Able to access funds for release time - Protected time enabled nurses to be able to do QI work | - Lack of support - managers perceived as not being present or supportive - Managers not effective as other managers - Managers being new to the role - Shift work schedules created difficulty in engaging nurses at regular intervals in the QI work - Heavy workloads – no time to complete QI project tasks |
| Kakyo  (2017)  [43] | Nursing and Health Sciences | Australia | To gain an understanding of nurse manager's CQI experiences in rural hospitals in Uganda | Nurse managers: current employment as a nurse manager & at least 2 years of experience | Interpretive qualitative using semi-structured interviews | 11 nurse managers |  | Managers in rural healthcare settings used their role to prioritize QI activities, monitor the CQI process, and utilized in-service education to support CQI. They utilized their management style and leadership abilities to ensure successful CQI.  Four themes:   1. Prioritizing continuous quality improvement activities in the local context 2. Monitoring the process of CQI 3. Using education and training to support CQI 4. Barriers encountered | - • Set a professional example - Highly enthusiastic - Had expert skills in managing resources in resource-poor settings | - Aligned CQI strategies to reflect the health priority goals of UN - Tracked patient numbers using graphical presentation to monitor progress - Made progress reports to senior administration for feedback - Adopted training strategies such as workshops continuous medical education sessions | NR | - Managers did not receive formal management or leadership training - Low staffing levels - Inadequate resources - Lack of consumer participation in CQI |
| Kallas (2014)  [73] | Nurse Admin Q | USA | To identify the profile of an excellent nurse manager | Not defined | Cross sectional survey design using the LPI-Self survey. Managers participated in one of two NDNQI-RN surveys between 2009-2011. | 36 CNEs representing 40 hospitals and 233 managers (3 groups -ranked excellent or competent by CNE and/or responded to NDNQI-RN surveys) |  | Found statistically significant distinctions between nurse managers who are excellent and those who are competent as assessed by the 5 practices of Exemplary Leadership which together form the profile of an excellent nurse manager.  Group 1: Across all nurse manager ratings, (4) Enable others to act had the highest mean score followed by (1) Model the way, (5) Encourage the heart, (3) Challenge the process, and (2) Inspire a shared vision.  Group2: No statistically significant differences in the five practices.  Group 3: 4/5 practices of Exemplary Leadership were found to be statistically significant for excellent nurse managers than for competent nurse managers. (1) Model the way, (3) Challenge the process, (4) Enable others to act, (5) Encourage the heart. Across all manager ratings, (4) Enable others to act had the greatest mean scores for excellent and competent nurse managers. The practice of (2) Inspire a shared vision was not statistically significant and had the lowest mean score. |  | NR |  |  |
| Kislov 2016 [37] | Public Administration | UK | To examine the case of an inter-organizational collaborative research partnership using clinicians as formalized KBs across multiple sites where research evidence was to be implemented | KB defined as clinical professionals (nurses and OT) external to the organization in which they were brokering knowledge | single embedded case study using interviews and observational data | 57 research participants, including 9 managers, drawn from three projects & management team |  | Identified 3 strategies that hybrid professionals used to surmount challenges associated with knowledge brokering: (1) relying on additional boundary ‘bridges’ such as clinicians & managers to link them with wider professional and organizational groups; (2) by conforming to existing ways of doing things and being flexible & tailoring approaches; and (3) shifting from ‘facilitating’ to ‘doing. Suggest that successful local assimilation of tacit and explicit knowledge is only possible when this external knowledge is spread within the organization through internal knowledge brokering embedded in collective practice. Initiatives using designated boundary spanning roles could benefit from diversifying the pool of KBs to include managers, quasi-managerial professionals and professionals with formal managerial responsibilities. |  | KBs:   - Performing skills audits - Coordinating the patient journey between different providers - Verifying registration and case finding in primary care - Feeding back results to staff - Facilitating the changes in day to day clinical practice - Providing staff help with data searches, teamwork development and guideline use - Acting as a conduit for knowledge sharing between practices - Liaising between primary and secondary care - Searching, analyzing and presenting the evidence - Helping staff improve the management of chronic disease patients - Actively involved in face to face meetings for knowledge sharing | - Seen as clinically legitimate because of clinical expertise - Using additional boundary “bridges” to link with wider professional and organizational groups at different levels - Being flexible and making use of existing procedures rather than trying to impose their views - Linking knowledge they were brokering with the national performance management system | - Heavy involvement in implementation took attention away from facilitation and linkage and exchange - Staff not given protected time to work on project - Staff expressed little interest in implementation - Staff needed encouragement - If activities involved a radical change from usual routines or did not explicitly link with adopted performance indicators - KBs seen as lacking seniority and having limited influence within their own organizations |
| Kitson 2011 [68] | J Nsg Manage-ment | Australia | To describe the experiences of clinical nurse leaders, team members and service managers' experiences introducing a KT project in one acute care hospital | Not defined | Qualitative -semi-structured interviews | 14 clinical nursing leaders, 28 team members, 11 service managers (7 nursing & 4 medical) | Organization-wide initiative - The Older Person and Improving Care project to improve the care of older adults in seven clinical topics: nutrition, functional decline, continence, confusion, clinical assessment, wayfinding, pain management. Two clinical nursing leaders led 7 teams for the 7 topics with the goal of introducing clinical evidence into their own area and spread across other units in the organization from 2007-2009. | Clinical nursing leaders identified risks and anxieties associated with taking on an additional leadership role, whereas managers acknowledged the multiple pressures on the system and the need for local level innovation. Team members generally reported positive experiences.  5 Main Themes of Clinical Nursing Leaders Experiences:   - Task - Risks - Learning - Hopes and expectations - Fears/anxieties of delivery & non-delivery   4 main themes of Nursing & Medical Service Managers:   - Pressures on the service - Role/Identity - Innovation despite culture - Patient-centered care | - Increased confidence - Increased knowledge - A “can do "attitude” - Ability to work in effective interdisciplinary teams - A level of maturity & understanding about the complexity of the innovation task | - Focused on contextualization of local implementation project into the wider system effectiveness agenda - Identified areas of successful small-scale innovations where change was actively embraced, and staff were involved in the process | - Identified as being flexible and enthusiastic pillars in dealing with change and innovating to improve ways of delivering care - Way of bringing people together - Important mechanism around which staff could focus on fundamentals of care | - Too busy putting out fires - Poorly designed processes and systems - Resource issues impacting on capacity to provide desired standard of nursing care - Resistance to change –staff not being able to see the bigger picture - Very busy and complex health system - Managers dealing with multiple issues/ competing demands - Lack of cohesion and identity of working practices of different professional groups - Work practices siloed and limited collaboration across professional and unit boundaries - Personaliza-tion of problems, conflict between staff - Lack of technical skills and knowledge |
| Lalleman 2015 [49] | Advances in Nursing Science | Netherlands & USA | To explore nurse MMs supporting role behavior (support for nurses from Nurse MM) | Nurse Middle Managers (NMM) defined as:   - Registered nurse, having a middle management position in adult care unit of acute care hospital - Supervising a nursing unit between 20 and 40 beds - Positioned between the work floor and higher management - With first-line responsibilities regarding the supervision of care workers and management of finances and quality of care | Exploratory multisite ethnographic case study | 16 Nurse MMs from 4 different hospitals - 2 in Netherlands & 2 in USA |  | Study found that: NMMs habitus (second nature) are a set of 8 dispositions (way of being): caring, clinical, collegial, teaching, professional, scientific, administrative and control. Various dispositions were valued differently at the 4 hospitals. It was found a combination of a caring, clinical, and scientiﬁc disposition enhances NMMs’ capital hospitals. |  | - Used their clinical expertise to know the patients and their conditions on their units. - Saw patients and discussed patient care with physicians and nursing staff - Available at nursing station to respond to staff calls for assistance related to clinical issues. - Gave attention to team members, encouraging feedback being collegial and preserving a friendly atmosphere - Responsible and accountable for patient outcomes but it was found that NMMs preoccupied with caring for the staff instead of the patients could ultimately compromise quality of patient care. - Coached and instructed nursing staff and students on patient care plans - Provided feedback and asked reflective questions rather than ad hoc action - Participated in rounds and multidisciplinary meetings and had short visits to the unit |  |  |
| Ott 2009  [59] | Nurse Economics | USA | To describe the introduction of the Clinical Nurse Leader (CNL) role in a multi-site health care system | Clinical Nurse Leader: Masters prepared nurse generalist is prepared to deliver and direct evidence-based practice who assumes accountability for healthcare outcomes of a specific group of clients | Evaluation of introduction of CNL pilot project from 2004-2008 | 7 VA medical center sites |  | - RN hours per patient day increased from 3.76 to 4.07 - Per-operative cancellations rates due to patient related reasons dropped to 50% - Per-operative cancellations dropped from 84% to 53% - GI surgical no show and cancellation rates decreased from 30% to 14%. - Over 1-year sitter hours decreased from 647/month to 24/month & reflects potential savings of $10,234 for the facility - Pressure ulcer prevalence dropped from 12.5% to 4.2% - Falls per 1000 patient days decreased from 1.93 to 1.37 - Rate of compliance with discharge teaching started at 13% and rose to 100% compliance - Incidence of ventilator assisted pneumonia dropped from 21.7% to 8.7% - Restorative dining showed an 8% increase in participation in the first month | Care provider who remains at bedside | - Clinical consultant and mentor for direct care nurses - Care coordinator for specific group of patients/ families - Lateral integrator for patient driven care across the continuum - Quality/ process improvement expert in clinical microsystems - Leader and role model for cost efficient, care delivery systems |  |  |
| Ploeg 2010 [44] | Worldviews on EBNursing | Canada | To determine how nursing best practice champions influence diffusion of BPG recommendations | Not defined | Mixed method sequential triangulation. 2 phases: qualitative interview & quantitative survey | Phase 1: 23 champions  Phase 2: 191 champions & 41 administrators |  | Nurse champions have a multidimensional role (take on roles such as educator, facilitator, mentor, leader, policy developer and evaluator to diffuse a guideline), and may be well positioned to promote an evidence-based culture and respond to the needs of nurses and other healthcare professionals in adopting evidence based practices. |  | Nurse champions:   - Actively raised awareness about BPGs through the use of formal and informal education - Acted as a resource to support and mentor nurses - Being persuasive practice leaders at all committee levels - Participated in and leading teams - Tailored guideline implementation strategies to organizational context –adapted materials - Explored, audited, monitored best practices –explored the literature, audit clinical indicators - Monitored practice gaps in the organization - Used feedback strategies to reinforce positive patient outcomes from guideline implementation - Involved in developing organizational specific policies and documents |  |  |
| Schell 2013 [48] | Engineer-ing Mgmt J | USA | To understand the similarities & differences in engineering and nurse manager roles in healthcare process improvement | Clinical Nurse Leader: Masters prepared nurse who practices at the microsystem level to improve client outcomes | Multi- case study | 26 Nursing case studies |  | Both groups commonly serve in MM roles. Engineers tend to experience change management challenges based on a lack of understanding of the engineering toolset or systems thinking approach by healthcare providers. Nurses see challenges driven by a lack of understanding of the microsystem and patient care level. Engineering managers should recognize that the nurse manager can be a key partner in working to implement successful process improvements within healthcare organizations. Nurse managers can assist engineering managers in bridging the cultural differences between the two groups resulting from their roles. Those in middle management roles can have a meaningful impact on the success of any change effort. Nurse change challenge themes identified:   1. Macro-mandated change 2. Ineffective communication/confusion 3. Caught in the middle 4. Need for change rationale/evidence 5. Take creative action 6. Resistance/sabotage 7. Trust/distrust 8. Lack of incentives |  | CNL:   - Discovered a creative work around process - Located influential partners - Bridged management’s requirements and patients’ needs - Provided a unique perspective to what is working and what is not working on the ground |  | - Confusion regarding the change being implemented - Change mandated from senior management without any input from staff - Need for clarity, vision, and mission of the organization - Physicians not on-board - Lack of incentives to change |
| Schreiber 2015 [63] | Soc Science Med | USA | To describe the use of a KT program to improve knowledge and frequency of use of standardized outcome measures | KBs: local opinion leaders - colleagues who are influential and trustworthy | Case report with self-report surveys, knowledge assessment, and chart reviews | 17 physical therapists in a pediatric outpatient facility | Implementation of a KT program over 6 months that included identification of barriers, use of a knowledge broker, multiple workshop and practice sessions, on-line and hardcopy resources, and ongoing evaluation of KT program with dissemination of results to staff. | KT program led to improved knowledge & increased the frequency of use of standardized outcome measures. Knowledge scores significantly increased between baseline (54.1) and 8-month follow-up (81.8). Self-reported knowledge of testing and measurement significantly improved across all the 4 subdomains between baseline and 8 months follow up. Documented frequency of administration increased for all pediatric outcome measures after the initiation of the KT program and was sustained over the 8-month period. | - Strong understand-ing of clinical and organizational contexts - Strong research skills - Enthusiasm - Accessibility | - Acts as a link between clinicians and the research evidence - Helps staff interpret and use evidence during clinical decisions on an ongoing basis |  |  |
| Sellgren 2006 [74] | J Nsg Manage-ment | Sweden | To explore nursing leadership regarding what nurse managers and subordinates see as important and to explore subordinates' opinions of their nurse manager's performance | Nurse Manager:   - Responsibility for a budget and human resources & 10 subordinates or more - Been in charge for 6 months or more | Questionnaire to assess preferred leadership behavior in 3 dimensions: change, production & employee/ relation orientations | 77 nurse managers, 770 subordinates |  | Statistically significant differences in opinions of preferred leadership between managers & subordinates in the dimensions’ production and employee orientation (tasks & employee relations). Subordinates prefer managers with more clearly expressed leadership behavior than managers prefer and demonstrate. The three most common leadership profiles were: super leader 12 managers (23.1%), nine managers (17.3%) got low scores in all three dimensions were identified as invisible leaders. These managers were vague in their leadership profiles. Third most common profile 26 managers (50%) were middle of the road leaders. |  | NR |  |  |
| Shaw 2010 [52] | Work | Canada | To understand the barriers & facilitators in brokering knowledge to help injured workers make informed decisions about recovery & to support their transitions to return to work | Not defined | Exploratory qualitative - interviews and focus groups | 106 participants from 63 injured worker groups & 43 healthcare professionals |  | Findings showed interactive knowledge transfer processes used to help injured workers understand and use knowledge. It was found that participants lacked expertise in knowledge transfer. Barriers in transferring knowledge included: system barriers, a lack of accessible information, problems with variations in capacity, experience using information.  Two major themes:   1. Information accessibility & Use barriers – barriers experienced in transferring information to workers 2. Facilitating interactive knowledge transfer –reflects actions that informed, facilitated, and supported KT |  | - Explored and determined information needs - Invited workers to engage in information exchange - Developed a plan for regular feedback opportunities - Developed a social context to establish trust and rapport - Tailored information –matched approach with needs | KBs understand worker capacity and readiness to receive and use information | - Difficulty knowing when and how to use information - Limited resources - Lack of time and remuneration - Inconsistencies in the way information is shared and degree of collaboration among stakeholders - Lack of opportunities for interaction with one another for sharing approaches & knowledge - Little or no training in strategies to promote effective knowledge brokering and knowledge transfer with end users |
| Traynor 2014 [62] | Public Health | Canada | To investigate a KB intervention as a means of enhancing capacity for EIDM in public health | KBs: experienced public health practitioners with expertise in research methodology and the EIDM process | Qualitative analysis from a single mixed method (RCT with qualitative) + case study | RCT = 108 staff, Case study: Case A, Case B & Case C = 804 staff | RCT: Health departments were randomly allocated to one of three progressively more active KT interventions  Access to review-level evidence in an online registry  Tailored email messages with embedded links to evidence summaries of high-quality systematic reviews plus registry access  KB services (i.e. the ‘KB intervention’) plus registry access and tailored messages.  Case Study:  Conducted in partnership with three Ontario health departments to evaluate the effective-ness of a 22-month KT intervention implemented by KBs. | All participants interviewed across the two studies described their involvement with the KBs as a largely positive and helpful experience. The KB was called a mentor, an advocate, a go-between, champion, and cheerleader. Several themes were identified from the qualitative analysis across both studies (see themes). Overall quantitative findings from the RCT showed that tailored messages led to a significant increase in the number of programs and policies supported by research evidence (P < 0.01), while exposure to either the KB intervention or the online registry did not (P < 0.45). Impact of the KB intervention was found to be moderated by organizational research culture (e.g. value placed on research evidence in decision making). However, in subgroup analysis, health departments exposed to the KB intervention that rated the organization low on a 7-point Likert scale on culture for EIDM at baseline, had a statistically significant increase in the number of programs and policies supported by research evidence at follow-up.  Case Study Results illustrate that participants who worked closely with the KB showed a statistically significant change in knowledge and skill (average increase of 2.8 points out of a possible 36 points (95% CI 2.0 to 3.6, P <0.001)) from baseline. These participants also showed a statistically significant increase of 49% in EIDM behaviors from baseline (P < 0.05).  Several themes identified from the qualitative analysis across both studies:   - Enhancing capacity - Effective KB attributes - Optimal positioning and ways of working | KB attributes:   - Expertise in research methodology & public health - Approachability and patience - Comfort in dealing with people at multiple levels - Ability to gain trust - Ability to adjust style to the person with whom they are working - Strong communication skills - Knowledge of EIDM & information management - Ability to pick up new knowledge quickly | KBs:   - Provided initial and ongoing needs assessments - Remained up to date on new evidence and resources in relevant content areas (scanned the horizon) - Managed published and unpublished evidence, internal evaluation information (knowledge management) - Facilitated individual capacity development in EIDM - Facilitated and supported organizational change | - Neutrality of KB - Strong management support - KBs credibility - Respect for and trust in the KBs - KB reliably available for in person meetings - KB responsive and timely with support | - Feasibility & costs - Prioritization of EIDM - Workload & time constraints - Role Clarity |
| Urquhart  2018 [38] | Worldviews Evidence-B Nsg | Canada | To examine the role of MMs in implementation innovation & how they experience the process | MM= Employees supervised by organization’s top managers & who supervise frontline employees | Qualitative- grounded theory | N=15 MMs |  | MMs see themselves as responsible for making implementation happen. Carry out 5 roles- planner, coordinator, facilitator, motivator,  evaluator. Found 2 determinants of MM role in implementation: they perform many other roles and responsibilities, and they have limited decision-making power and must work within the parameters set by senior management |  | Planner- anticipate needs, plan; accordingly, Coordinator- organize, connect all the components; Facilitator-create the environment to enable successful implementation; Motivator- promote buy-in and provide emotional support;  Evaluator- monitor progress & impacts | NR | NR |
| Urquhart 2019 [24] | J Health Services Res & Policy | Canada | To identify influences on MMs’ commitment to innovation implementation | MM= Employees supervised by organization’s top managers & who supervise frontline employees | Qualitative using grounded theory | N= 15 MMs |  | MM s contemplate 2 issues in terms of their commitment to implementation: 1. Ease of implementation, 2. Potential benefit for the patients. Identify factors that influence MMs commitment to innovation implementation: available resources, fit with setting, stakeholder buy-in |  |  | Available resources, fit with setting, stakeholder buy-in |  |
| Uvhagen  2018 [66] | BMC Health Serv Research | Sweden | To analyze how managers, interpret and make sense of a large top down implementation initiative & implications this has for implementation process | NR | Exploratory Qualitative | N=8 managers |  | The way managers interpreted and made sense of the task was influenced by: how they perceived the different parts of the initiative, how they perceived themselves, and how they perceived the resources available for the initiative. Implementation in health care is complex. Not the content that influences the implementation but rather how the parts are perceived by the manager. |  |  | - Early and on-going dialogue about how the implementation task is perceived by the manager responsible for implementation | - Inadequate resources |
| Waring 2013 [54] | Social Science Med | UK | To identify and compare embedded KBs working within and between organizational teams, departments, units, especially clinical and managerial groups | Risk Manager (RM): formal delegated responsibility for developing, communicating and managing hospital policies & procedures for patient safety.  Risk Officers (RO): supported formal hospital reporting and learning procedures within clinical departments. Clinical Leaders (CL): formal administrative or leadership responsibilities for service planning, delivery, coordination and governance. Professional Leaders (PL): clinicians or other specialists appointed or volunteered for professional leadership roles | 2 stage exploratory study - observation and interviews | 19 Risk Managers, Risk Officers, Clinical Leaders, & Practice Leaders in three large teaching hospitals |  | Suggests KBs occupying hybrid organizational roles, such as MMs and Clinical Leaders, often best positioned to support knowledge sharing & learning because of their 'ambassadorial' type position & legitimacy to participate in multiple communities through dual-directed relationships.  Variations in formal role, location and relationships shape how 4 types of intra-organizational brokers share and support the use of knowledge across organizational and occupational boundaries & were highly adept & effective at brokering knowledge across professional & managerial boundaries.  Found 3 linked themes:   1. Significance of the broker’s structural position within a hospital -ambassorial brokers tended to have a hybrid role that blurred traditional boundaries that enabled brokers to more easily mediate knowledge and cultural differences; 2. Brokers varied according to their role within the organization - formal brokering seen in more RM, ROs admin roles. Informal brokering in CL, PL roles. CL, PL more effective in brokering & relied more on relational qualities; 3. Observed brokers varied in type of knowledge shared & types of activities performed to support learning. Formal brokers (RM, ROs) tended to rely on documented or explicit knowledge. Those CL, PLs with legitimate membership & participation within clinical practice communities were able to experience, understand & share their "know-how". |  | PL:   - Promoting learning or change within uniprofession-al and department groups - Participated in department/ hospital committees related to patient safety - Communica-ted professional updates and guidance through emails, weekly briefings   CL:   - Provided clinicians with opportunity to reflect on safety events - Provided open door policy for staff to make sense of their tacit experiences - Gathered documents, reviewed evidence, organized interviews during formal incidents - Translated safety recommendations into department procedures - Translated policies into local practice context   RO:   - Helped staff complete reports and forms correctly - Supported the work of the CL with updates - Met regularly with CLs to give verbal updates on safety events - Provided weekly safety updates across departments   RM:   - Used secondary data to identify incident trends and events - Made initial contact with CLs to gather further evidence - Initiated a case meeting to analyze evidence - Provided analysis and recommendations |  |  |
| Warshawsky 2013  [58] | Nurse Admin | USA | To understand effects of nurse managers’ practice environments on staff & patient outcomes | Nurse managers defined as first-line registered nurse managers with 24-hour responsibility and accountability for the overall supervision of all registered nurses and other healthcare providers working in designated acute patient care areas. | secondary content analysis of qualitative data from 2 cross sectional studies using electronic surveys | 127 nurse managers |  | Nurse managers rated 8 domains of nurse managers' practice environments from most to least influence:   1. culture of generativity (time to develop front line staff), 2. effective nurse manager-unit staff relationships, 3. culture of meaning 4. empowering admin leaders create a culture of patient safety, 5. fair & manageable workload, 6. constructive nurse manager- director relationships, 7. adequate budgeted resources, 8. collegial relationships between nurse managers & physicians.   Nurse managers in this analysis reported characteristics of the practice environment that limit their role effectiveness. Four themes emerged as to managers' needs to practice:   1. Manageable workloads 2. Adequate time on assigned patient care areas 3. Directors who empower them to make decisions 4. Administrative leaders who create a collaborative culture that supports safe patient care |  | - Accountable 24/7 for their patient care areas. - Responsible for large number of employees as well as staff education and regulatory compliance for department. - Effectively translates the organization’s mission, vision, values, and strategic plan to the front-line staff to create a culture of meaning - Partners with their front-line staff to achieve quality patient outcomes. | - Positive working relationships and trust between managers and their staff - Feeling supported and valued by the senior leadership team - Ability to be creative, problem solve, and implement new things on unit that are recognized by upper nursing management - Directors that facilitate growth and development by providing constructive performance feedback | - Time away from unit - Scope of responsibilities - Time to adequately coach staff - Significant portions of workday absorbed by non-nursing functions (budgeting, facilities, trouble shooting) - Inadequate time to nurture and support frontline staff - Large numbers of direct reports - Disconnect with upper administration and the bedside - Multiple conflicting priorities - Staffing issues - Dissatisfaction with workload or work life balance - Unable to disengage from their work - Lack of support from director - Hospital purchasing contracts interfered with obtaining appropriate material resources - Dissatisfaction with disparities in expectations between nursing staff and physician staff. - Hostile interactions between physicians and nurses |
| Williams 2012 [45] | J Adv Nursing | UK | To explore and explain how an intermediary program in infection control practice worked in one hospital | Intermediaries defined as: nurses operating under the direction of the nurse for infection control with clinical responsibilities | Mixed methods case study | 9 participants (manager, matron, nurses, healthcare support worker, intermediary) |  | Specific actions of intermediaries can impact staff behavior. For frontline staff, intermediary presence triggered a modification in behavior. Different reactions were noted from the intermediaries’ high level of physical presence in clinical areas, the facilitative approaches they used to give feedback and the specific teaching strategies they employed to meet frontline staff needs. The specific intermediary actions uncovered in this study were contingent on the prevailing systems for performance management, organizational commitment and efforts in clinical areas to foster a collegiate environment. |  | - Gave thoughtful feedback on performance - Developed relationships in clinical areas - Built rapport and trust with staff - Provided educational programs - Collaborated with clinical leads and infection control team - Provided surveillance of clinical areas | - Organizational commitment & investment - High visibility of the intermediary - Making teaching relevant to local context - Tailored education for staff | NR |
| Wilson 2013 [57] | J Nsg Manage-ment | USA | To demonstrate how the Clinical Nurse Leaders (CNL) contribute to improving quality of care | Clinical nurse leader: Masters prepared advanced generalist nurse who assumes accountability for healthcare outcomes of a specific group of clients | Concept analysis: anecdotal description of activities and impact in a single hospital. Evaluated effectiveness of role with measures of clinical outcomes, financial savings and case studies. | 8 CNLs in a 637-bed tertiary care facility & community hospital |  | CNL role implementation demonstrated the following clinical outcomes:   - Decrease the readmission rate in one physician practice 11.3% - Decrease in Code Blue Events 88% - Decrease in deterioration events 73% - Increase in Rapid Response Team use 133% - Decrease in blood stream infections 52% - Decrease in the use of intermediate care bed post-operatively 50% - Decrease in overall LOS 39% - Decrease in post-procedure 67% - Increase in vaccination for pneumonia patients 138% - Increase in influenza vaccine given hospital wide 66.5% - Increase in pneumococcal vaccine given hospital wide 53% - Decrease in pressure ulcers in intensive care unit 50% - Decrease in pressure ulcers on a medical unit 81.8% - Decrease time from patient booked to patient arrival onto unit 59% - Increase in patient education given29.4 % - Decrease in pain on a 10-point scale 56 % - Decrease length of recovery unit stay 42%   Financial outcomes:   - Costs related to a decrease in equipment and staff time $150 000 - Costs related to decreased ventilator days $1 000 000 Costs related to decreased LOS $170 000 - Costs related to decreased in post-procedure LOS $ 139 000 - Costs related to decreased recovery room and hospital LOS $1 200 000 - Total= $2.7 M |  | - Members of organizational decision-making groups for strategic planning - Promoted interdisciplinary collaboration - Developed partnerships with patients, family and the community - Engage front-line staff in problem-solving - Partnered with physicians as co-leaders of clinical interdisciplinary teams - Initiated hospital wide rounds to coordinate care and share best practices - Served as authoritative contact for those initiatives - Developed tools, algorithms and order sets to support interdisciplinary care delivery - By working with front-line staff, identified problems and conducted root cause analysis - Share information with staff, patients, family and community partners - Utilized established networks to improve collaboration among staff - Participated in multiple research projects as primary and co-investigators | NR | NR |

NR=Not Reported
