# Supplementary material for: The roles, activities and impacts of middle managers who function as knowledge brokers to improve care delivery and outcomes in healthcare organizations: a critical interpretive synthesis
Source: BMC Health Serv Res. 2022 Jan 2;22:11. doi: 10.1186/s12913-021-07387-z (PMC8722036; doi:10.1186/s12913-021-07387-z)
Supplement: Supplementary file 5 — Additional file 5. Quality appraisal tools and findings [file 12913_2021_7387_MOESM5_ESM.docx]

# Quality appraisal checklist findings

**Standards for Quality Improvement Reporting Excellence****(SQUIRE 2.0)** [34]

| **First Author & Date** | | **Bullock 2012 [56]** | **Ott 2009 [59]** | **Wilson 2012 [57]** |
| --- | --- | --- | --- | --- |
|  | **Title and Abstract** | | | |
| **1** | **Title** | Good | Good | Good |
| **2** | **Abstract** | Well described | Well described | Well described |
|  | **Introduction** *Why did you start?* | | | |
| **3** | Problem Description | Well described | Well described | Well described |
| **4** | **Available Knowledge** | Well described | Yes | Well described |
| **5** | Rationale | Well described | Well described | Well described |
| **6** | **Specific Aim** | Well described | Well described | Well described |
|  | **Methods** *What did you find?* | | | |
| **7** | Context | Good | Good | Good |
| **8** | Interventions | Good | Well described | Good |
| **9** | **Study of the interventions** | Good | Very well described | Good |
| **10** | **Measures** | Well described | Well described | Good |
| **11** | **Analysis** | Well described | Well described | Well described |
| **12** | **Ethical Considerations** | Not stated | Conflict of Interest addressed | Good |
|  | **Results** *What did you find?* | | | |
| **13** | **Results** | Well described | Well described | Well described |
|  | **Discussion** *What does it mean?* | | | |
| **14** | **Summary** | Yes | Yes | Yes |
| **15** | **Interpretation** | Well described | Yes | Well described |
| **16** | **Limitations** | Yes | Yes | Yes |
| **17** | **Conclusions** | Yes | Yes | yes |
|  | **Other information** |  |  |  |
| **18** | **Funding** | Addressed | Not stated | Addressed |

**Good Reporting of a Mixed Methods Study (GRAMMS) checklist** [32]

| **First author**  **& date** | **Birkin 2015 [47]** | **Bradley 2006 [55]** | **Currie 2014 [8]** | **Dobbins**  **2018 [16]** | **Donahue 2013 [50]** | **Ploeg 2010 [44]** | **Traynor 2014 [62]** | **Williams 2012 [45]** |
| --- | --- | --- | --- | --- | --- | --- | --- | --- |
| **GUIDELINE Section: page** |  |  | | | | | | |
| Describe justification for using a mixed methods approach to the research question | Design: pg. 161 | Design: pg. 325  Discussion: strengths & limitations: pg. 334 | Methods pg. 54-56 | Methods  pg. 2-3 | Methods: pg. 28-29 | Design: pg. 240  Study Limitations: pg. 247-8 | Methods: pg. 534-35 | Design: pg. 917 Limitations: pg. 923 |
| Describe the design in terms of purpose, priority, and sequence of methods | Design: pg. 161 | Methods: pg. 325-27 | Methods pg. 54-56 | Methods  pg. 2-3 | Methods: pg. 28-29 | Design: pg. 240 | Methods: pg. 534-35 | Design: pg. 917 |
| Describe each method in terms of sampling, data collection and analysis | Methods pg. 162-164 | Methods: pg. 325-327 | Methods pg. 54-56 | Methods  pg. 3-4 | Methods: pg. 28-29 | Methods pg. 240-42 | Methodspg. 534-35 | Data collection: pg. 917 |
| Describe where integration has occurred, how it has occurred and who has participated in it | Design: pg. 161 | _______ | ______ | _______ | _______ | Methods pg. 240-42 | ____ | _____ |
| Describe any limitation of one method associated with the present of the other method | Discussion pg. 166 | Strengths & limitations: pg. 334 | Limitations: pg. 119-120 | Limitations: pg. 13 | Discussion pg. 32 | Study Limitations: pg. 247-48 | Discussion: pg. 539-41 | Limitations: pg. 923 |
| Describe any insights gained from mixing or integrating methods | Discussion: pg. 166-67 | Discussion: pg. 334 | Conclusion: pg. 117-120 | Discussion: pg. 11-13 | Discussion: pg. 32 | Discussion: pg. 248-49 | Discussion: pg. 539-41 | Discussion: pg. 922-23 |

**BI Critical Appraisal Checklist for Quasi-Experimental Studies (non-randomized experimental studies)** [35]

Reviewer F. Boutcher Date August 2018

Author **Balding**  Year **2005**

Record Number [60]

|  | Yes | No | Unclear | Not  applicable |
| --- | --- | --- | --- | --- |
| 1. Is it clear in the study what is the ‘cause’ and what is the ‘effect’ (i.e., there is no confusion about which variable comes first)? | 🗹 | □ | □ | □ |
| 2. Were the participants included in any comparisons similar? | 🗹 | □ | □ | □ |
| 3. Were the participants included in any comparisons receiving similar treatment/care, other than the exposure or intervention of interest? | 🗹 | □ | □ | □ |
| 4. Was there a control group? | 🗹 | □ | □ | □ |
| 5. Were there multiple measurements of the outcome both pre- and post-the intervention/exposure? | 🗹 | □ | □ | □ |
| 6. Was follow up complete and if not, were differences between groups in terms of their follow up adequately described and analyzed? | 🗹 | □ | □ | □ |
| 7. Were the outcomes of participants included in any comparisons measured in the same way? | 🗹 | □ | □ | □ |
| 8. Were outcomes measured in a reliable way? | 🗹 | □ | □ | □ |
| 9. Was appropriate statistical analysis used? | 🗹 | □ | □ | □ |

Overall appraisal: Include 🗹 Exclude □ Seek further info □

Comments (Including reason for exclusion) _______________________

**Critical Appraisal of a Questionnaire Study** [33]

| **First Author & Date** | Birkin 2016 [64] | Fryer 2018 [67] | Kallas 2014  [73] | Schreiber  2015 [63] | Sellegren  2006 [74] | Warshawsky  2013 [58] |
| --- | --- | --- | --- | --- | --- | --- |
| **Appraisal Questions** |  | | | | | |
| What information did the researchers seek to obtain? Was there a clear research question, and was this important and sensible? Was a questionnaire the most appropriate research design for this question, what design might have been more appropriate? | Yes | Yes | Yes | Yes | Yes | Yes |
| What was the sampling frame and was it sufficiently large and representative? Did all participants in the sample understand what was required of them, and did they attribute the same meaning to the terms in the questionnaire? | Yes | Yes | Yes | Yes | Yes | Yes |
| Were there any existing measures (questionnaires) that the researchers could have used? If so, why was a new one developed and was this justified? | No.  Yes, was justified | Yes | Yes | N/A | N/A | Yes |
| Were the views of consumers sought about the design, distribution, and administration of the questionnaire? | Yes | Not stated | Yes | Not stated | Not stated | Not stated |
| What claims for reliability and validity have been made, and are these justified? Did the questions cover all relevant aspects of the problem in a non-threatening and non-directive way? Were open-ended (qualitative) and closed-ended (quantitative) questions used appropriately? Was a pilot version administered to participant’s representative of those in the sampling frame, and the instrument modified accordingly? | Yes | Yes | Yes | Yes | Yes | Not stated |
| What claims for validity have been made, and are they justified? (In other words, what evidence is there that the instrument measures what it sets out to measure?) | Yes | Yes | Yes | Yes | Yes | Not stated |
| What claims for reliability have been made, and are they justified? (In other words, what evidence is there that the instrument provides stable responses over time and between researchers?) | N/A | Yes | Yes | Yes | Yes | Not stated |
| Was the title of the questionnaire appropriate and if not, what were its limitations? | Yes | Yes | Yes | Yes | Yes | Yes |
| What formats did the questionnaire take, and were open and closed questions used appropriately? | Yes | Not available | Not available | Not available | Not available | Yes |
| Were easy, non-threatening questions placed at the beginning of the measure and sensitive ones near the end? | Yes | Not available | Not available | Not available | Not available | Not available |
| Was the questionnaire kept as brief as the study allowed? What was the response rate and have non-responders been accounted for? | Yes | Yes | Yes | Yes | Yes | Yes |
| Did the questions make sense, and could the participants in the sample understand them? Were any questions ambiguous or overly complicated? | Yes | Not stated | Not stated | Yes | Yes | Not stated |
| Did the questionnaire contain adequate instructions for completion—e.g., example answers, or an explanation of whether a ticked or written response was required? | Yes | Yes | Yes | Not stated | Yes | Not stated |
| Were participants told how to return the questionnaire once completed? | Yes | Not stated | Yes | Not stated | Not stated | Not stated |
| Did the questionnaire contain an explanation of the research, a summary of what would happen to the data, and a thank you message? | Not stated | Not stated | Not stated | Not stated | Not stated | Not stated |
| Was the questionnaire adequately piloted in terms of the method and means of administration, on people who were representative of the study population? | Yes | Yes | N/A | Yes | N/A | Yes |
| How was the piloting exercise undertaken? What details are given? | Yes | Yes | N/A | Yes | N/A | N/A |
| In what ways was the definitive instrument changed as a result of piloting? | N/A | Not clear | N/A | Not clear | N/A | N/A |
| What was the sampling frame for the definitive study and was it sufficiently large and representative? | Yes | Yes | Yes | No | Yes | Yes |
| Was the instrument suitable for all participants and potential participants? Did it take account of the likely range of physical/mental/cognitive abilities; language/literacy, understanding of numbers/scaling, and perceived threat of questions or questioner? | Yes | Yes | Yes | Yes | Yes | Yes |
| How was the questionnaire distributed? | In person | electronically | In person | At meetings | By mail | electronically |
| How was the questionnaire administered? | Self- administered | By email | Self- administered | Self- administered | Self- administered | Self-administered |
| Were the response rates reported fully, including details of participants who were unsuitable for the research or refused to take part? | Yes | Yes | Yes | Yes | Yes | Yes |
| Have any potential response biases been discussed? | No | No | No | No | Yes | No |
| What sort of analysis was carried out and was this appropriate? (e.g., correct statistical tests for quantitative answers, qualitative analysis for open ended questions) | Yes | Yes | Yes | Yes | Yes | Yes |
| What measures were in place to maintain the accuracy of the data, and were these adequate? | Adequate | Good | Adequate | Adequate | Adequate | Adequate |
| Is there any evidence of data dredging—that is, analyses that were not hypothesis driven? | No | No | No | No | No | No |
| What were the results and were all relevant data reported? | Yes | Yes | Yes | Yes | Yes | Yes |
| Are quantitative results definitive (significant), and are relevant non-significant results also reported? | N/A | Yes | Yes | Yes | Yes | N/A |
| Have qualitative results been adequately interpreted (e.g., using an explicit theoretical framework), and have any quotes been properly justified and contextualized? | Yes | Yes | Yes | Yes | N/A | Yes |
| Was the analysis appropriate (e.g. statistical analysis for quantitative answers, qualitative analysis for open-ended questions) and were the correct techniques used? Were adequate measures in place to maintain accuracy of data? | Yes | Yes | Yes | Yes | Yes | Yes |
| What do the results mean and have the researchers drawn an appropriate link between the data and their conclusions? | Yes | Yes | Yes | Yes | Yes | Yes |
| Have all relevant results (‘significant’ and ‘non-significant’) been reported? Is there any evidence of ‘data dredging’ (i.e., analyses that were not ‘hypothesis driven’)? | Yes | Yes | Yes | Yes | Yes | Yes |
| Have the researchers drawn an appropriate link between the data and their conclusions? | Yes | Yes | Yes | Yes | Yes | Yes |
| Overall rating | **++** | **++** | **+** | **+** | **++** | **+** |

Use this checklist can improve the evaluation of a questionnaire study.

| Have the findings been placed within the wider body of knowledge in the field (e.g., via a comprehensive literature review), and are any recommendations justified? |
| --- |
| Can the results be applied to your organization? |
| Conflicts of interest are declared. |
| Rate the overall methodological quality of the study, using the following as a guide:  **High quality (++)**: Majority of criteria met. Little or no risk of bias.  **Acceptable (+):** Most criteria met. Some flaws in the study with an associated risk of bias.  **Low quality (-):** Either most criteria not met, or significant flaws relating to key aspects of study design.  **Reject (0):** Poor quality study with significant flaws. Wrong study type. Not relevant to guideline. |

**Standards for Reporting Qualitative Research (SRQR)** [31]

| **First Author & Date** | **Bradley**  **2003 [53]** | **Chang**  **2013 [69]** | **Currie**  **2015 [40]** | **Dobbins**  **2019 [17]** | **Engle**  **2017 [51]** | **Fleiszer**  **2015**  **[41]** | **Girard**  **2013 [61]** | **Hitch**  **2014 [39]** | **Jeffs**  **2013**  **[46]** | **Jeffs**  **2016 [42]** | **Kakyo**  **2017**  **[43]** | **Kislov**  **2016 [37]** | **Kitson**  **2011 [68]** | **Lalleman**  **2015**  **[49]** | **Schell**  **2013 [48]** | **Shaw**  **2010 [52]** | **Urquhart**  **2018**  **[38]** | **Urquhart**  **2019**  **[24]** | **Uvhagen**  **2018 [66]** | **Waring**  **2013 [54]** |
| --- | --- | --- | --- | --- | --- | --- | --- | --- | --- | --- | --- | --- | --- | --- | --- | --- | --- | --- | --- | --- |
| **Page/line no(s).** |  | | | | | | | | | | | | | | | | | | | |
| **Title & Abstract** | Pg. 15 | Pg.36 | Pg. 793 | Pg. 1 | Pg. 14 | Pg.309 | Pg. 60 | Pg. 389 | Pg. 222 | Pg. 367 | Pg. 244 | Pg. 472 | Pg. 542 | Pg. E1 | Pg. 33 | Pg. 89 | Pg. 414 | Pg.91 | Pg. 1 | Pg. 79 |
| **Introduction,**  **Problem formulation, Purpose, or research questio**n | Pg. 16, line 29 | Pg.37-38 | Pg. 794-796 | Pg. 2 | Pg. 15-16 | Pg.310 | Pg.60-63 | Pg. 389-90 | Pg. 222-23 | Pg. 367-368 | Pg. 244-45 | Pg. 472 | Pg. 543 | Pg. E1-3 | Pg. 33-35 | Pg. 89-92 | Pg. 414-15 | Pg.91-92 | Pg. 1-2 | Pg. 79-81 |
| **Methods** | Pg. 16, line 53 | Pg. 38 | Pg. 796-98 | Pg. 2-4 | Pg. 16 | Pg. 310-312 | Pg.63 | Pg. 390-91 | Pg. 223-26 | Pg. 368-69 | Pg. 245-46 | Pg. 476-78 | Pg. 544 | Pg. 3 | Pg. 35-37 | Pg. 91 | Pg. 415-16 | Pg.92 | Pg. 2-4 | Pg. 81-82 |
| **Research paradigm** | Not stated | Not stated | Not stated | Not stated | Not stated | Not stated | Not stated | Not stated | Not stated | Not stated | Not stated | Not stated | Pg. 544 | Not stated | Not stated | Not stated | Pg.415 | Pg. 92 | Not stated | Not stated |
| **Researcher characteristics & reflexivity** | Not stated | Not stated | Not stated | Not stated | Not stated | Not stated | Pg. 63-64 | Pg. 390-91 | Not stated | Not stated | Not stated | Not stated | Not stated | Not stated | Not stated | Not stated | Pg. 415-16 | Pg. 92-93 | Not stated | Not stated |
| **Context** | Pg. 16 | Pg.38 | Pg. 794 | Pg. 2 | Pg. 16 | Pg. 310 | Pg. 63 | Pg. 390-91 | Pg. 223 |  | Pg. 245 | Pg. 476 | Pg. 544 | Pg. E3 | Pg. 35 | Pg. 92-93 | Pg. 415 | Pg.92 | Pg. 2 | Pg. 81-82 |
| **Sampling strategy** | Pg. 16 | Pg.38 | Pg. 797 | Pg. 2-3 | Pg.16-17 | Pg. 311 | Pg. 63 | Pg. 390-91 | Pg. 223 | Pg. 368 | Pg. 245 | Pg. 476 | Pg.544 | Pg. E3 | Pg. 35 | Pg. 92 | Pg.415 | Pg.92 | Pg. 2-3 | Pg. 81-82 |
| **Ethical issues pertaining to human subjects** | Ethics approval not stated | Pg. 38 | Ethics approval not  stated | Pg. 2 | Ethics approval not stated | Pg. 316 | Pg. 64 | Pg. 390 |  |  | Pg. 245 | Ethics approval not  stated | Pg. 544 | Pg. E4 |  | Ethics approval not  stated | Pg.415 | Pg.92 | Pg. 9 | Ethics approval not  stated |
| **Data collection methods** | Pg. 17 | Pg.38 | Pg. 796-98 | Pg. 3-4 | Pg.16 | Pg. 312 | Pg. 63 | Pg. 391 | Pg. 223-24 | Pg. 368 | Pg. 245 | Pg. 477 | Pg. 544 | Pg. E4 | Pg. 35-37 | Pg. 92-93 | Pg.415 | Pg.92 | Pg. 3 | Pg. 81-82 |
| **Data collection instruments & technologies** | Pg. 17-18 | Pg. 38 | Pg. 797 | Pg. 3-4 | Pg.16 | Pg. 311 | Pg. 63 | Pg. 391 | Pg. 223-24 | Pg. 368 | Pg. 245 | Pg. 476 | Pg. 544 | Pg. E4-5 | Pg. 36 | Pg. 92-93 |  | Pg.92-93 | Pg. 3 | Pg. 81-82 |
| **Units of study** | Pg. 17-18 | Pg.39 | Pg. 797-98 | Pg. 2 | Pg.17 | Pg. 311-12 | Pg. 63 | Pg. 391 | Pg. 226 | Pg. 369 | Pg. 246 | Pg. 478 | Pg.545 | Pg. E3 | Pg. 36 | Pg. 92-93 | Pg. 416 | Pg.93 | Pg. 3 | Pg. 82-84 |
| **Data processing** | Pg. 17-18 | Pg. 38 | Pg. 797-98 | Pg. 4 |  | Pg. 311-12 | Pg. 63 | Pg. 391 | Pg. 224 | Pg. 368 | Pg. 245-46 | Pg. 478 | Pg. 544 | Pg. E4 | Pg. 36 | Pg. 93 | Pg. 415-16 | Pg.93 | Pg. 4 | Pg. 82 |
| **Data analysis** | Pg. 17 | Pg. 38-39 | Pg. 797-98 | Pg. 4 | Pg.16- 17 | Pg. 311-12 | Pg. 63 | Pg. 391 | Pg. 224 | Pg. 368-69N | Pg. 245-46 | Pg. 477-78 | Pg. 544 | Pg. E5 | Pg. 36 | Pg. 93 | Pg.415 | Pg.93 | Pg. 4 | Pg. 82 |
| **Techniques to enhance trustworthiness** | No | No | No | No | No | Pg.312 | Pg. 63-64 | Pg. 391 | No | No | Pg. 245 | Pg. 478 | No | Pg. E5 | No | No | Pg.416 | Pg.93 | Pg. 4 | No |
| **Synthesis and interpretation & Links to empirical data** | Pg.19-25 | Pg.39-40 | Pg. 798-807 | Pg. 4-5 | Pg. 18-24 | Pg.312-15 | Pg. 64-65 | Pg. 392-94 | Pg. 226-28 | Pg. 369-70 | Pg.246-48 | Pg. 478-482 | Pg. 545-552 | Pg. E5-12 | Pg. 37-39 | Pg. 94-98 | Pg. 416-19 | Pg.93-96 | Pg. 4-6 | Pg. 82-84 |
| **Integration with prior work, implications, transferability contribution(s) to the field** | Pg. 25-26 | Pg. 40-41 | Pg. 807-9 | Pg. 5-6 | Pg.24-26 | Pg.315-316 | Pg.65-66 | Pg. 394-96 | Pg. 228-29 | Pg. 370-71 | Pg. 248 | Pg. 482-85 | Pg. 552-554 | Pg. E12-15 | Pg. 39-40 | Pg. 98-100 | Pg. 419-21 | Pg. 97-98 | Pg. 7 | Pg. 84-85 |
| **Limitations** | Pg. 26 | Pg. 41 | Not stated | Not stated | Pg.26 | Pg. 316 | Pg. 66 | Pg. 395 | Pg. 229 | Pg. 371 | Pg. 248 | Not stated | Pg.553 | Pg. E14 | Pg. 40 | Not stated | Pg. 421 | Pg.98 | Not stated | Not stated |
| **Conflicts of interest** | Not stated | Pg.42 | Not stated | Pg. 7 | Pg.14 | Pg. 316 | Pg. 66 | Pg.396 | Pg. 222 | Pg. 367 | Not stated | Not stated | Pg. 554 | Pg. E1 | Not stated | Not stated | Pg. 421 | Pg.98 | Pg. 9 | Not stated |
| **Funding** | Pg. 27 | Not stated | Pg.809 | Pg. 7 | Pg.27 | Pg. 316 | Pg.66 | Not stated | Not stated | Pg. 367 | Pg. 249 | Not stated | Pg. 554 | Pg. E1 | Not stated | Pg. 100 | Pg. 421 | Pg.98 | Pg. 9 | Not stated |
